# Supplementary material for: Rapid isolation of potent SARS-CoV-2 neutralizing antibodies and protection in a small animal model
Source: bioRxiv. 2020 May 15:2020.05.11.088674. Preprint. [Version 2] doi: 10.1101/2020.05.11.088674 (PMC7263516; doi:10.1101/2020.05.11.088674)
Supplement: Supplement 1 [file media-1.pdf]

SUPPLEMENTARY MATERIAL

Materials and Methods
Tables S1 – S2
Figures S1 – S12
Supplementary References (16 – 25)

Materials and Methods

<u>HeLa-hACE2 Stable Cell Line</u>

HeLa-hACE2 and A549-hACE2 cells were generated through transduction of human ACE2 lentivirus. pBOB-hACE2 construct was co-transfected into HEK293T cells along with lentiviral packaging plasmids pMDL, pREV, and pVSV-G (Addgene) by Lipofectamine 2000 (Thermo Fisher Scientific, 11668019) according to the manufacturer's instructions. Supernatants were collected 48 h after transfection, then were transduced to pre-seeded HeLa or A549 cells. 12 h after transduction, stable cell lines were collected, scaled up and stored for neutralization assay.

<u>Virus generation</u>

Vero E6 cells (ATCC CRL-1586) were plated in a T225 flask with complete DMEM (Corning 15-013-CV) containing 10% FBS, 1X PenStrep (Corning 20-002-CL), 2 mM L-Glutamine (Corning 25-005-CL) overnight at 37C 5% CO2. The media in the flask was removed and 2 mL of SARS-CoV-2 strain USA-WA1/2020 (BEI Resources NR-52281) in complete DMEM was added to the flask at an MOI of 0.5 and was allowed to incubate for 30 minutes at 34˚C 5% CO2. After incubation, 30 mL of complete DMEM was added to the flask. The flask was then placed in a 34°C incubator at 5% CO2 for 5 days. On day 5 post infection, the supernatant was harvested and centrifuged at 1,000× g for 5 minutes. The supernatant was filtered through a 0.22 µM filter and stored at -80˚C.

<u>SARS-CoV-2 focus reduction neutralization test (FRNT)</u>

HeLa-ACE2 cells were plated in 12 µL complete DMEM at a density of 2×103 cells per well. In a dilution plate, plasma or mAb was diluted in series with a final volume of 12.5 µL. 12.5 µL of SARS-CoV-2 was added to the dilution plate at a concentration of 1.2E4 pfu/mL. The dilution plate was covered and incubated at 34°C 5% CO2 for 1h. After incubation, the media remaining on the 384-well cell plate was removed and 25µL of the virus plasma/mAb mixture was added to the 384-well cell plate. The plate was incubated for 20hat 34°C 5% CO2. After incubation, the plate was fixed with 25 µL of 8% Formaldehyde for 1h at 34°C 5% CO2. The plate was then washed three times with 100 µL of 1×XPBS 0.05% tween. 12.5 µL of human polyclonal sera diluted 1:500 in Perm/Wash buffer (BD Biosciences 554723) were added to the plate and incubated at RT for 2h. The plate was washed three times with 100 µL of 1×XPBS 0.05% tween. 12.5 µL of Peroxidase goat anti-human Fab (Jackson Scientific) were diluted 1:200 in Perm/Wash buffer then added to the plate and incubated at RT for 2h. The plate was then washed three times with 50 µL of 1×X PBS 0.05% tween. 12.5 µL of Perm/Wash buffer was added to the plate then incubated at RT for 5 min. The Perm/Wash buffer was removed and TrueBlue peroxidase substrate was immediately added (Sera Care 5510-0030). Infected cell non-linear regression curves were analyzed using Prism 8 software to calculate EIC_{50} values.

<u>Pseudovirus (PSV) Assay</u>

MLV-gag/pol and MLV-CMV-Luciferase plasmids were co-transfected with full-length or truncated SARS-CoV-2 and SARS-CoV-2 plasmid, respectively, with transfection reagent Lipotransfectmine 2000 in HEK293T cells. After 48 h of transfection, supernatants containing pseudotyped virus were collected and frozen at -80°C for long-term storage. Serially diluted plasma or mAbs were incubated with pseudovirus at 37°C for 1 h, then transferred onto HeLa-hACE2 cells in 96-well plates at 10,000 cells/well (Corning, 3688). After 48 h of incubation, supernatants were removed, HeLa-hACE2 cells were then lysed in luciferase lysis buffer (25mM Gly-Gly pH 7.8, 15mM MgSO4, 4 mM EGTA, 1% Triton X-100). Luciferase activity was measured by adding Bright-Glo (Promega, PR-E2620) according to the manufacturer's instructions. Plasma or mAbs were tested in duplicate wells. Neutralization ID50 or IC_{50}

titers were calculated using “One-Site Fit LogIC$_{50}$” regression in GraphPad Prism 8.0.

Cohort information

De-identified PBMC and plasma were kindly provided through the “Collection of Biospecimens from Persons Under Investigation for 2019-Novel Coronavirus Infection to Understand Viral Shedding and Immune Response Study” UCSD IRB# 200236. Protocol was approved by the UCSD Human Research Protection Program.

Whole Virus ELISA

High binding plates (Corning 3700) were coated with 12.5 µL of Galanthus Nivalis Lectin (GNL; Vector Laboratories L-1240-5) at 10 µg/mL and incubated overnight at 4°C. The GNL was removed and 12.5 µL of SARS-CoV-2 was added to the plate at a concentration of 2E6 pfu/mL then incubated for 24 hours at 4°C. 12.5 µl of 8% Formaldehyde was added to a final concentration of 4% then incubated at RT for 1 hour. The plate was then washed three times with 100 µL of 1x PBS supplemented with 0.05% tween. 50 µL of 3% BSA were added to the plate and incubated at RT for 2 hours. The BSA was removed and 12.5 µL of plasma or mAb diluted in series was added to the plate then incubated at RT for 1.5 hours. The plate was then washed three times with 100 µL of 1x PBS supplemented with 0.05% tween. 12.5 µL of alkaline phosphatase conjugated goat anti-human Fc antibody (Jackson Immunoresearch 109-055-098) diluted 1:2000 was added to the plate and incubated for 1 hour at RT. The plate was then washed three times with 100 µL of 1x PBS supplemented with 0.05% tween. 12.5 µL of phosphatase substrate (SIGMA-ALDRICH S0942) were added to the plate. Non-linear regression curves were analyzed using Prism 8 software to calculate EC50 values.

Plasmid construction for full-length and recombinant soluble proteins

To generate full-length SARS-CoV-1 (1255 amino acids; GenBank: AAP13567) and SARS-CoV-2 (1273 amino acids; GenBank: MN908947) spike plasmids, genes were synthesized from GeneArt (Life Technologies), cloned into mammalian expression vector phCMV3 (Genlantis, USA) using PstI and BamH restriction sites. Soluble S ectodomain protein SARS-CoV-1 (residue 1-1190) and SARS-CoV-2 (residue 1-1208) encoding plasmids were constructed by PCR amplifications and Gibson assembly cloning into vector phCMV3. To stabilize soluble S proteins in prefusion state and to improve trimerization, double proline substitutions in the S2 subunit, replacement of a furin cleavage site in SARS-CoV-2 (residues 682–685), and S2 cleavage site in SARS-CoV-1 (residues 664–667) with “GSAS” and incorporation of a C-terminal T4 fibritin trimerization motif, as described previously [16,17]. An HRV-3C protease cleavage site, a 6X HisTag, and an AviTag spaced by GS-likers were added to the C-terminus to aid purification strategies. To generate gene fragments encoding SARS-CoV-2 N-terminal domain-NTD (residue 1-290), receptor-binding domain-RBD (residue 332-527), RBD-SD1 (residue 320-591), and RBD-SD1-2 (residue 320-681) subdomains, PCR-amplifications were carried out from the SARS-CoV-2 plasmid and gene fragments were cloned in frame with the original secretion signal or the Tissue Plasminogen Activator (TPA) leader sequence. A similar design strategy was used to construct the SARS-CoV-1-RBD (residue 319-513) gene encoding plasmid.

Flow cytometry based cell surface SARS-CoV-1/CoV-2 spike binding assay

Binding of mAbs/sera to the HEK293T cell-surface expressed SARS-CoV-1 and SARS-CoV-2 spikes was performed as described previously [18]. Briefly, HEK293T cells were transfected with plasmids encoding full-length SARS-CoV-1 or SARS-CoV-2 spikes and incubated for 36-48 hours at 37 °C. Post incubation cells were trypsinization to prepare single cell suspension and were distributed into 96-well plates. 50ul/well of 3-fold serial titrations of mAbs starting at 10 µg/mL or serum samples starting at 1:30 dilution were added to transfected cells. The Abs were incubated with cells for 1 hr on ice. The plates were washed twice in FACS buffer (1X PBS, 2% FBS, 1mM EDTA) and stained with 50ul/well of 1:200 dilution of R-phycoerythrin (PE)-conjugated mouse anti-human IgG Fc antibody (SouthernBiotech) and 1:1000 dilution of Zombie-NIR viability dye (BioLegend). After another two washes, stained cells were analyzed using flow cytometry (BD Lyrics cytometers), and the binding data were generated by calculating the percent (%) PE-positive cells for antigen binding using FlowJo 10 software. CR3022, a SARS-CoV-2 spike binding antibody, and unrelated antibody, DEN3, were respectively positive and

negative controls for the assay.

Protein expression and purification

To express the soluble S ectodomain protein SARS-CoV-1, SARS-CoV-2 and their truncated protein versions, protein-encoding plasmids were transfected into FreeStyle293F cells (Thermo Fisher) at a density of approximately 1 million cells/mL. For large scale production, we mixed 350ug plasmids with 16 mL transfectagro™ (Corning) in a conical tube and filtered with 0.22um Steriflip™ Sterile Disposable Vacuum Filter Units (MilliporeSigma™). In another conical tube, we added 1.8mL 40K PEI (1 mg/mL) into 16 mL transfectagro™ and mixed briefly. The premixed 40K PEI- transfectagro™ solution was gently poured into the filtered plasmid solution. The solution was thoroughly mixed by inverting the tube several times. The mixture rested at room temperature for 30 mins and was poured into 1L FreeStyle293F cell culture. After 5 days, the cells were removed from the supernatant by centrifuging at 3500 rpm for 15mins. The supernatant was filtered in a glass bottle with the 0.22um membrane and kept in 4-degree storage before loading into the columns. The His-tagged proteins were purified with the HisPur Ni-NTA Resin (Thermo Fisher). To eliminate the nonspecific binding proteins, each column was washed with at least 3 bed volumes of wash buffer (25 mM Imidazole, pH 7.4). To elute the purified proteins from the column, we loaded 25mL of the elution buffer (250mM Imidazole, pH 7.4) at slow gravity speed (~4 sec/drop). By using Amicon tubes, we buffer exchanged the solution with PBS and concentrated the proteins. The proteins were further purified by size-exclusion chromatography using Superdex 200 (GE Healthcare). The selected fractions were pooled and concentrated again for the further use.

Recombinant Protein ELISAs

6x-His tag monoclonal antibody (Invitrogen, UA280087) was coated onto high-binding 96-well plates (Corning, 3690) at 2 μg/mL overnight at 4 °C. After washing, plates were blocked with 3% BSA in PBS for 1h. Then his-tag recombinant RBD and S protein were captured at 1μg/mL in 1% BSA and incubated for 1h at RT. After washing, serially diluted mAbs were added into wells and incubated for 1h at RT. Detection was measured with alkaline phosphatase-conjugated goat anti-human IgG Fcg (Jackson ImmunoResearch) at 1:1000 dilution for 1h. After the final wash, phosphatase substrate (Sigma-Aldrich) was added into wells. Absorption was measured at 405 nm.

12.5 ul of 6x-His tag monoclonal antibody (Invitrogen UA280087) were coated onto high binding plates at 2μg/mL overnight at 4°C. The plate was washed three times with 100 ul of 1xPBS/0.05% Tween. The recombinant RBD and S protein derived from SARS-CoV-1 and SARS-CoV-2 were diluted with 1xPBS/1%BSA to final concentrations of 2 μg/mL or 5 μg/mL and then coated onto the plate and incubated at RT for 2h. The plate was washed three times with 100 ul of 1xXPBS/0.05% Tween and then blocked with 100 ul of 3% BSA at RT for 2 hours and then the BSA was removed. Either serially diluted plasma samples or isolated mAbs were added onto the plate and incubated for 1.5 hours at RT. Wells were then incubated with secondary anti-human IgG Fcγ antibody diluted at 1:2000 and incubated at RT for 1 hour. The plate was then washed three times with 100 μL of 1xPBS/0.05% tween. 12.5uL of phosphatase substrate (SIGMA-ALDRICH S0942) were added to the plate and optical density (OD) was measured at 405 nm. Plasma or mAbs were tested in duplicate or triplicate wells. Non-linear regression curves were analyzed using Prism 8 software to calculate IC_{50} values.

Isolation of SARS-2 S-specific mAbs

Sorting of antigen-specific memory B cells was performed as previously described [19,20]. The process was adapted for high-throughput such that each step could be performed in a 96-well format. Fluorescent-labeled antibodies recognizing cell surface markers were purchased from BD Biosciences. AVI-tagged SARS-2 S and RBD proteins were produced, purified, labeled with biotin (Avidity), and coupled to streptavidin-AF647, streptavidin-AF488 (Thermo Fisher), and streptavidin-BV421 (BD Biosciences), as previously described [20] at 2:1 and 4:1 molecular ratio respectively 30 min prior to staining. Cells were first labeled with antibodies for surface markers together with probes (200 nM final) for 30min in sort buffer (PBS 1% FBS, 2.5M EDTA, 25mM Hepes) on ice. Cells were then stained with the Live/Dead Fixable Aqua Dead Cell Stain (Thermo Fisher) for 15 minutes on ice according to the

manufacturer's instructions. Single antigen-specific (S+ and RBD+) memory B cells (CD3-CD4-CD8-CD14-CD19+IgD-IgG+) were sorted into individual empty wells of a 96-well plate using a BD FACSAria Fusion sorter. Plates were immediately sealed and stored at-80°C.

cDNA was generated from cells sorted using Superscript IV Reverse Transcriptase (Thermo Fisher), dNTPs (Thermo Fisher), random hexamers (Gene Link) and Ig gene-specific primers in a lysis buffer containing Igepal (Sigma), DTT and RNAseOUT (Thermo Fisher). Nested PCR amplification of heavy- and light-chain variable regions was performed using Hot Start DNA Polymerases (Qiagen, Thermo Fisher), and previously described primer sets [21,22]. Second round PCR primers were modified to include additional nucleotides overlapping with the expression vectors. PCR efficiency was assessed using 96w E-gels (Thermo Fisher). Paired wells picked individually, re-arrayed into new 96w plates and cloned in-frame into expression vectors encoding the human IgG1, Ig kappa or Ig lambda constant domains using the Gibson Assembly Enzyme mix (New England BioLabs) according to manufacturer instructions. Ligation reactions were transformed into DH5a competent E-coli, transferred into 1mL Plasmid+ media (Thomson Instrument Company) supplemented with antibiotic and grown overnight at 37C under agitation. The next day the cultures were used to inoculate duplicate cultures before being lysed for plasmid DNA extraction using NucleoSpin 96 miniprep kit (Macherey-Nagel, Takara). Cloned heavy- and light-chain variable regions were sequenced (Genewiz) and subsequently analyzed using the IMGT (International ImMunoGeneTics Information System, www.imgt.org) V-quest webserver [23].

Flow cytometry-based cell surface ACE2 binding inhibition assay.

Monoclonal antibody inhibition of SARS-CoV-2 S or RBD binding to cell surface hACE2 was performed by flow cytometry as follows. Purified mAbs were mixed with biotinylated SARS-CoV-2 S or RBD in the molar ratio of 4:1 on ice for 1h. In the meantime, HeLa-ACE2 cells were washed once with DPBS then detached by incubation with DPBS supplemented with 5 mM EDTA. The detached HeLa-ACE2 cells were washed and resuspended with FACS buffer (2% FBS and 1 mM EDTA in DPBS). 0.5 million Hela-ACE2 cells were added to mAb/S or RBD mixture and incubated at 4°C for 0.5 h. HeLa-ACE2 cells were then washed once with FACS buffer, resuspended FACS buffer with 1 μg/mL streptavidin-AF647 (Thermo, S21374) and incubated for another 0.5 h. After washing, HeLa-ACE2 cells were resuspended in FACS buffer in the presence of 2 μg/mL propidium iodide (Sigma, P4170-100MG) for live/dead staining. HeLa and HeLa-ACE2 cells stained with SARS-CoV-2 S or RBD alone were used as background and positive control separately. The AF647 mean fluorescence intensity (MFI) was determined from the gate of singlet and PI negative cells. The percentage of ACE2 binding inhibition was calculated using the following equation.

Antibody expression and purification

Antibodies HC and LC constructs were transiently expressed with the Expi293 Expression System (Thermo fisher). After 4 days, 24-deep well culture supernatants were harvested to be directly tested for binding and neutralization. Selected mAbs showing neutralizing activity in the HTP screening were re-expressed in small to medium scale cultures using individual colonies plasmid DNA, and IgG purified on Protein A sepharose (GE Healthcare).

Epitope binning by bio-layer interferometry

Neutralizing and non-neutralizing mAbs were binned into epitope specificities using an Octet RED384 system. 50-100 nM of HIS-tagged S or RBD protein antigens were captured using anti-Penta-HIS biosensors (18-5120, Molecular Devices). After antigen loading for 5 min, a saturating concentration of mAb (100 μg/mL) was added for 10 min. Competing concentrations of mAbs (25 μg/mL) were then added for 5 min to measure binding in the presence of saturating antibodies. All incubation steps were performed in 1x PBS.

Surface Plasmon Resonance Methods

SPR measurements were collected using a Biacore 8K instrument at 25°C. All experiments were carried out with a flow rate of 30 μL/min in a mobile phase of HBS-EP+ [0.01 M HEPES (pH 7.4), 0.15 M NaCl, 3 mM EDTA, 0.0005% (v/v) Surfactant P20]. Anti-Human IgG (Fc) antibody (Cytiva) was immobilized to

a density of ~7000-10000 RU via standard NHS/EDC coupling to a Series S CM-5 (Cytiva) sensor chip. A reference surface was generated through the same method.

For conventional kinetic/dose-response, listed antibodies were captured to ~50-100 RU via Fc-capture on the active flow cell prior to analyte injection. A concentration series of SARS-CoV-2 RBD was injected across the antibody and control surface for 2 min, followed by a 5 min dissociation phase using a multi-cycle method. Regeneration of the surface in between injections of SARS-CoV-2 RBD was achieved with a single, 120 s injection of 3M MgCl2. Kinetic analysis of each reference subtracted injection series was performed using the BIAEvaluation software (Cytiva). All sensorgram series were fit to a 1:1 (Langmuir) binding model of interaction.

A SPR assay was also used to assess the competition between SARS-CoV-2 RBD and ACE2 for binding to CC12.1 [24,25]. CC12.1 was captured to the surface of 3 flow cells to ~100 RU via Fc-capture. SARS-CoV-2 RBD was injected to each flow cell at a concentration of 50 nM to establish a basal level of SARS-CoV-2 RBD binding. This concentration was held constant for the competition experiments, which were carried out by varying the ACE2 concentration over eight points from 800 to 6.25 nM. To calculate residual SARS-CoV-2 RBD binding, the sensorgram responding to the corresponding ACE2 injection alone was subtracted from the SARS-CoV-2 RBD plus ACE2 injection series. The average response for the 5 s preceding the injection stop was plotted against the concentration of ACE2 and fit to a dose-response inhibition curve by nonlinear regression [log(inhibitor) vs. response – variable slope (4 parameters)] using GraphPad Prism. Regeneration between injections was carried out as noted above.

Animal Study

SARS-CoV-2 infection of 8-week old Syrian hamsters was achieved through the intranasal instillation of 10^6 total PFU per animal in 100 μl of PBS. Animals weights were obtained during the study as a measure of disease progression. Treatment groups included the intraperitoneal injection of varying doses of monoclonal antibody. After 12-h, serum was obtained to quantify mAb titers and animals were infected as described above. At day-5 post-infection, lungs were harvested for analysis. Research protocol was approved and performed in accordance with Scripps Research IACUC Protocol #20-0003.

Viral load measurements

Viral RNA was isolated from lung tissue and subsequently amplified and quantified in a RT-qPCR reaction. Lung tissue was extracted at day 5 post infection. The lung tissue was divided into sections approximately 100-300mg in size. Samples were placed in 1mL of TRIzol-LS reagent (Invitrogen). Samples for virus load were then subjected to tissue homogenization using disposable pestles in 15mL conical tubes (Corning). Tissue homogenates were then spun down to remove any remaining cellular debris and the supernatant was added to a RNA purification column (Qiagen). Purified RNA was eluted in 80uL of DNase-, RNase-, endotoxin-free molecular biology grade water (Millipore) and quantified using a nanodrop (Thermo Fisher). RNA was then subjected to reverse transcription and quantitative PCR using the CDC's N1 primer sets (Forward 5'-GAC CCC AAA ATC AGC GAA AT-3'; Reverse 5'-TCT GGT TAC TGC CAG TTG AAT CTG-3') and a double-quenched (ZEN/Iowa Black FQ) and fluorescently labeled (FAM) probe (5'-FAM-ACC CCG CAT TAC GTT TGG TGG ACC-BHQ1-3') (Integrated DNA Technologies) on an BioRad CFX96 Real-Time instrument. For quantification, a standard curve was generated by diluting 1×1010 RNA copies SARS-CoV-2 genome/mL, 10-fold in water (Virapur). Every run utilized eight, tenfold serial dilutions of the standard. SARS-CoV-2-positive and -negative samples were included.

Table S1. Cohort Demographics.

	PCR confirmed?	First blood collect date	Symptom onset (date)	Days post symptom onset	Age	Gender	Disease severity	Past medical history
**CC01**	Yes	13-Feb	9-Feb	4	51	F	Moderate	None
**CC04**	Yes	16-Feb	6-Feb	10	56	F	Mild	None
**CC05**	Yes	14-Feb	3-Feb	11	53	M	Moderate	None
**CC06**	Yes	13-Mar	5-Mar	8	59	M	Severe	Other
**CC08**	Yes	23-Mar	9-Mar	14	62	M	Severe	None
**CC09**	Yes	26-Mar	1-Mar	25	44	F	Mild	None
**CC10**	Yes	26-Mar	4-Mar	22	45	M	Mild-to-moderate	Asthma
**CC11**	Yes	26-Mar	1-Mar	25	43	F	Mild	None
**CC12**	Yes	26-Mar	1-Mar	25	39	F	Mild	None
**CC13**	Yes	26-Mar	1-Mar	25	48	F	Mild	None
**CC18**	Yes	31-Mar	8-Mar	23	48	F	Mild	None
**CC20**	No	31-Mar	Unknown	-	59	M	Mild	None
**CC21**	Yes	26-Mar	15-Mar	11	50	M	Severe	HIV, HTN
**CC22**	Yes	2-Apr	27-Mar	6	43	M	Critical	None
**CC23**	Yes	2-Apr	15-Mar	18	72	F	Moderate	CKD, CAD
**CC24**	Yes	7-Apr	14-Mar	24	32	M	Mild	None
**CC25**	Yes	7-Apr	11-Mar	27	42	M	Mild	None

Abbreviations: HLD, hyperlipidemia; HTN, hypertension; HCQ, hydroxychloroquine

Table S2. Antibody sequence analysis. Clonal families are highlighted in shaded colors.

	Sequence characterisitics			
**mAb ID**	**VH Gene**	**% SHM (nt)**	**VL Gene**	**% SHM (nt)**
**CC12.1**	IGHV3-53*01	1.05	IGKV1-9*01	1.08
**CC12.2**	IGHV3-53*01	0.70	IGKV3-20*01	3.55
**CC12.3**	IGHV3-53*01	1.40	IGKV3-20*01	0.36
**CC12.4**	IGHV1-2*04	1.74	IGLV2-8*01	1.74
**CC12.5**	IGHV1-2*02	4.86	IGLV2-14*01	3.47
**CC12.6**	IGHV1-2*02	4.17	IGLV2-14*01	1.74
**CC12.7**	IGHV1-2*02	7.99	IGLV2-14*01	6.60
**CC12.8**	IGHV1-2*02	3.12	IGLV2-14*01	1.04
**CC12.9**	IGHV1-2*02	3.47	IGLV2-14*01	1.39
**CC12.10**	IGHV1-2*02	4.51	IGLV2-14*01	4.17
**CC12.11**	IGHV1-2*02	3.47	IGLV2-14*01	1.74
**CC12.12**	IGHV1-2*02	3.47	IGLV2-14*01	2.08
**CC12.13**	IGHV3-53*01	0.35	IGKV1-33*01	3.23
**CC12.14**	IGHV3-21*01	3.47	IGKV2-30*01	1.70
**CC12.15**	IGHV3-48*03	1.04	IGLV1-40*01	1.04
**CC12.16**	IGHV3-33*01	1.74	IGLV3-21*02	1.08
**CC12.17**	IGHV3-30*03	1.39	IGLV3-21*03	1.79
**CC12.18**	IGHV1-46*01	0.00	IGLV6-57*01	1.03
**CC12.19**	IGHV3-23*04	1.04	IGLV3-21*02	1.79
**CC12.20**	IGHV3-30*03	1.04	IGLV1-47*01	1.05
**CC12.21**	IGHV1-24*01	1.39	IGLV1-44*01	0.70
**CC12.22**	IGHV1-24*01	1.04	IGKV3-15*01	0.00
**CC12.23**	IGHV4-39*01	0.69	IGLV3-25*03	0.36
**CC12.24**	IGHV3-30*03	1.04	IGKV1-39*01	0.72
**CC12.25**	IGHV3-23*04	0.35	IGLV1-44*01	2.46
**CC12.26**	IGHV5-51*01	0.35	IGLV7-43*01	1.04
**CC12.27**	IGHV1-2*02	1.04	IGLV2-23*01	0.35

Table S3. Hamster protection study summary.

Animal ID	CC12.1 Dose (μg)	Weight (g) at Day 0	Weight (g) at Day 1	Weight (g) at Day 3	Weight (g) at Day 4	Weight (g) at Day 5	Weight (g) at Day 6	Lung sample mass (mg)	Virus load (absolute)	Virus load per mg (VL/mg)	CC2.1 serum titer at Day 0 (μg/mL)	CC2.1 serum titer at Day 5 (μg/mL)
**1-334**	Ctrl	124	120	114	108	106		132	47381442133	358950319	0.00	0
**1-335**	Ctrl	109	100	98	94	93		166.6	2079731197	12483381	0.00	0
**1-338**	Ctrl	122	121	116	111	110		98.1	3929842857	40059560	0.00	0
**1-432**	Ctrl	131	126	120	114	112		248.4	952477443	3834450	0.00	0
**1-436**	Ctrl	120	118	108	103	102		202.8	1097002828	5409284	0.00	0
**1-433**	Ctrl	120	118	110	108	104		144.2	29037705468	201371050	0.00	0
**1-440**	2000	130	122	145	142	141		139.2	22203736	159510	46.99	97
**1-451**	2000	134	128	130	135	138		137.8	23367181	169573	28.46	68
**1-437**	2000	124	117	128	132	131		77.7	1758266	22629	20.77	135
**1-447**	2000	109	100	110	114	118		180	59773323	332074	49.67	0
**1-449**	2000	108	100	102	104	104		163.8	7562491	46169	29.95	75
**1-448**	2000	120	110	118	122	124		134.7	8504808	63139	29.36	98
**1-443**	500	119	110	120	121	123		39.3	7908498	201234	27.19	31
**1-427**	500	143	131	144	146	150		58.2	1731959	29759	19.92	42
**1-431**	500	120	110	112	114	116		155.7	111573372	716592	12.38	63
**1-429**	500	119	110	118	120	120		56.5	2427923	42972	18.89	42
**1-428**	500	98	90	94	96	98		66.3	12872027	194148	26.27	49
**1-441**	500	122	116	120	121	124		164.4	17194913	104592	38.80	42
**1-452**	125	130	130	126	122	121		135.7	9227879	68002	28.42	1
**1-445**	125	120	110	106	106	104		145.5	74616429	512828	15.04	1
**1-456**	125	90	92	90	88	87		135.4	96077317	709581	14.29	2
**1-454**	125	130	122	124	123	124		142.6	8874684	62235	5.16	2
**1-453**	125	128	120	115	114	118		188.4	2369242	12576	6.83	3
**1-455**	125	120	111	108	104	104		165.9	11500369	69321	4.20	6
**1-442**	31	110	100	98	95	94		113.3	146999226	1297434	9.82	0
**1-454**	31	118	102	106	106	103		60.8	230749502	3795222	3.50	0
**1-446**	31	110	100	98	96	94		145.8	7505018	51475	9.49	0
**1-443**	31	119	110	107	102	101		50	1264877867	25297557	6.67	0
**1-444**	31	120	101	100	97	94		65.8	385355044	5856460	1.44	0
**1-445**	31	100	90	86	84	84		65.2	1077752020	16529939	25.30	0
**1-458**	8	127	110	112	103	102		126.6	247726739	1956767	0.11	0
**1-459**	8	120	111	100	94	96		219.1	368023811	1679707	0.00	0
**1-457**	8	118	110	103	100	97		244.3	16935521	69323	0.29	0
**1-460**	8	100	96	92	87	85		104.4	1153435020	11048228	0.06	0
**1-463**	8	110	102	97	92	94		143.9	822113391	5713088	0.00	0
**1-461**	8	120	115	112	108	104		83	2813485933	33897421	0.12	0

Animal	CC12.26 Dose (μg)	Weight (g) at Day 0	Weight (g) at Day 1	Weight (g) at Day 3	Weight (g) at Day 4	Weight (g) at Day 5	Weight (g) at Day 6	Lung sample mass (mg)	Virus load (absolute)	Virus load per mg (VL/mg)	CC2.26 serum titer at Day 0 (μg/mL)
**26-422**	Ctrl	103		100	97		96				0.00
**26-410**	Ctrl	110		100	96		93				0.00
**26-425**	Ctrl	120		110	110		102				0.00
**26-423**	Ctrl	112		110	104		105				0.00
**26-424**	Ctrl	90		88	82		78				0.00
**26-426**	Ctrl	108		100	100		93				0.00
**26-414**	2000	100		100	100		96				94.69
**26-415**	2000	130		126	126		123				88.06
**26-408**	2000	128		126	121		119				88.13
**26-416**	2000	122		118	110		110				105.13
**26-413**	2000	120		118	118		112				70.32
**26-412**	2000	110		100	98		96				34.93
**26-409**	500	120		118	106		102				38.02
**26-403**	500	120		118	110		110				34.76
**26-404**	500	130		120	110		104				28.39
**26-402**	500	110		102	100		101				42.84
**26-406**	500	110		114	100		100				19.64
**26-405**	500	120		110	109		110				0.34
**26-388**	125	100		100	108		110				6.83
**26-390**	125	120		119	112		108				6.01
**26-391**	125	119		116	110		107				7.01
**26-439**	125	100		100	96		93				6.58
**26-389**	125	110		104	102		108				4.46
**26-387**	125	138		130	123		118				0.00
**26-411**	31	124		121	110		108				2.26
**26-417**	31	100		98	92		90				2.30
**26-421**	31	100		100	94		95				0.00
**26-419**	31	100		100	92		89				2.08
**26-420**	31	112		110	104		103				0.83
**26-418**	31	133		132	120		115				1.17
**26-407**	8	120		110	110		107				0.00
**26-393**	8	101		100	98		96				0.25
**26-394**	8	100		99	90		87				0.35
**26-396**	8	118		110	108		105				0.00
**26-395**	8	123		122	118		114				0.13
**26-392**	8	124		120	110		106				0.16

Table S4. Hamster protection study statistics. Each group was compared to the Den3 IgG control group

			CC12.1 protection experiment													
			IgG Day 0		Weigh Day 0		Weigh Day 1		Weigh Day 3		Weigh Day 4		Weigh Day 5		Viral Load Day 5	
Statistical test		Ab Dose (µg)	Summary	Adjusted P-Value	Summary	Adjusted P-Value	Summary	Adjusted P-Value	Summary	Adjusted P-Value	Summary	Adjusted P-Value	Summary	Adjusted P-Value	Summary	Adjusted P-Value
Grouped Parametric	One-way ANOVA	2000	****	<0.0001	ns	0.9957	ns	0.0972	**	0.0014	****	<0.0001	****	<0.0001	*	0.0274
		500	****	<0.0001	ns	0.9957	ns	0.0704	*	0.0319	****	<0.0001	****	<0.0001	*	0.0274
		125	*	0.0318	ns	0.9957	ns	0.5244	ns	0.4496	ns	0.2054	*	0.0269	*	0.0274
		31	ns	0.0923	ns	0.7037	***	0.0005	ns	0.2663	ns	0.3002	ns	0.2835	*	0.0274
		8	ns	0.9834	ns	0.8946	ns	0.0795	ns	0.3494	ns	0.2054	ns	0.2309	*	0.0274
Grouped Non-Parametric	Kruskall-Wallis	2000	****	<0.0001	ns	>0.9999	ns	0.6064	ns	0.1275	*	0.0192	*	0.0128	**	0.0025
		500	**	0.0011	ns	>0.9999	ns	0.0884	ns	0.2931	ns	0.0709	*	0.0477	**	0.0074
		125	*	0.0377	ns	>0.9999	ns	>0.9999	ns	>0.9999	ns	>0.9999	ns	>0.9999	**	0.0041
		31	ns	0.1111	ns	0.33	***	0.0007	ns	0.6242	ns	>0.9999	ns	>0.9999	ns	>0.9999
		8	ns	>0.9999	ns	>0.9999	ns	0.7306	ns	>0.9999	ns	>0.9999	ns	>0.9999	ns	>0.9999
Individual Parametric	t-test	2000	****	<0.0001	ns	0.9753	*	0.021	**	0.0051	****	<0.0001	****	<0.0001	ns	0.1131
		500	****	<0.0001	ns	0.9008	**	0.006	**	0.0012	****	<0.0001	****	<0.0001	ns	0.1134
		125	**	0.008	ns	0.8502	ns	0.6122	ns	0.4247	ns	0.0784	*	0.0179	ns	0.1135
		31	*	0.0219	ns	0.0863	**	0.0012	*	0.0163	ns	0.1784	ns	0.185	ns	0.1435
		8	ns	0.0541	ns	0.3115	ns	0.0504	ns	0.1005	ns	0.0784	ns	0.0524	ns	0.145
Individual Non-Parametric	Mann-Whitney	2000	**	0.0022	ns	>0.9999	*	0.0303	**	0.0043	**	0.0022	**	0.0022	**	0.0022
		500	**	0.0022	ns	0.5152	*	0.0238	**	0.0043	**	0.0022	**	0.0022	**	0.0022
		125	**	0.0022	ns	0.9264	ns	0.6667	ns	0.8506	ns	0.132	*	0.0152	**	0.0022
		31	**	0.0022	ns	0.0519	**	0.0043	**	0.0043	ns	0.2403	ns	0.1688	ns	0.132
		8	ns	0.0606	ns	0.29	*	0.039	ns	0.2403	ns	0.0693	ns	0.0866	ns	0.0931

			CC12.23 protection experiment													
			IgG Day 0		Weigh Day 0				Weigh Day 3		Weigh Day 4		Weigh Day 5			
		Ab Dose (µg)	Summary	Adjusted P-Value	Summary	Adjusted P-Value			Summary	Adjusted P-Value	Summary	Adjusted P-Value	Summary	Adjusted P-Value		
Grouped Parametric	One-way ANOVA	2000	****	<0.0001	ns	0.4367			ns	0.5798	ns	0.5035	ns	0.4976		
		500	**	0.0018	ns	0.4367			ns	0.5798	ns	0.7427	ns	0.9187		
		125	ns	0.8462	ns	0.6359			ns	0.4409	ns	0.4521	ns	0.1817		
		31	ns	0.9735	ns	0.6359			ns	0.1595	ns	0.9931	ns	0.9139		
		8	ns	0.9831	ns	0.6359			ns	0.5798	ns	0.9115	ns	0.9139		
Grouped Non-Parametric	Kruskall-Wallis	2000	****	<0.0001	ns	0.5146			ns	>0.9999	ns	>0.9999	ns	0.2503		
		500	**	0.0033	ns	0.6082			ns	>0.9999	ns	0.8312	ns	>0.9999		
		125	ns	0.0769	ns	>0.9999			ns	0.3718	ns	>0.9999	ns	0.5597		
		31	ns	0.4173	ns	>0.9999			ns	0.0878	ns	>0.9999	ns	>0.9999		
		8	ns	>0.9999	ns	>0.9999			ns	>0.9999	ns	>0.9999	ns	>0.9999		
Individual Parametric	t-test	2000	****	<0.0001	ns	0.1028			ns	0.2721	ns	0.1468	ns	0.0785		
		500	**	0.0015	ns	0.0549			ns	0.5508	ns	0.1866	ns	0.9123		
		125	***	0.0008	ns	0.332			ns	0.1307	ns	0.2308	ns	0.1449		
		31	**	0.0036	ns	0.5568			*	0.0159	ns	0.9877	ns	0.4424		
		8	ns	0.2431	ns	0.2652			ns	0.3764	ns	0.6137	ns	0.5128		
Individual Non-Parametric	Mann-Whitney	2000	**	0.0022	ns	0.119			ns	0.329	ns	0.2403	ns	0.0931		
		500	**	0.0022	ns	0.0758			ns	0.3268	ns	0.0693	ns	0.9848		
		125	*	0.0152	ns	0.5974			ns	0.1255	ns	0.3095	ns	0.2403		
		31	*	0.0152	ns	0.8463			*	0.0173	ns	0.9675	ns	0.3095		
		8	ns	0.0606	ns	0.329			ns	0.2576	ns	0.9654	ns	0.3939		

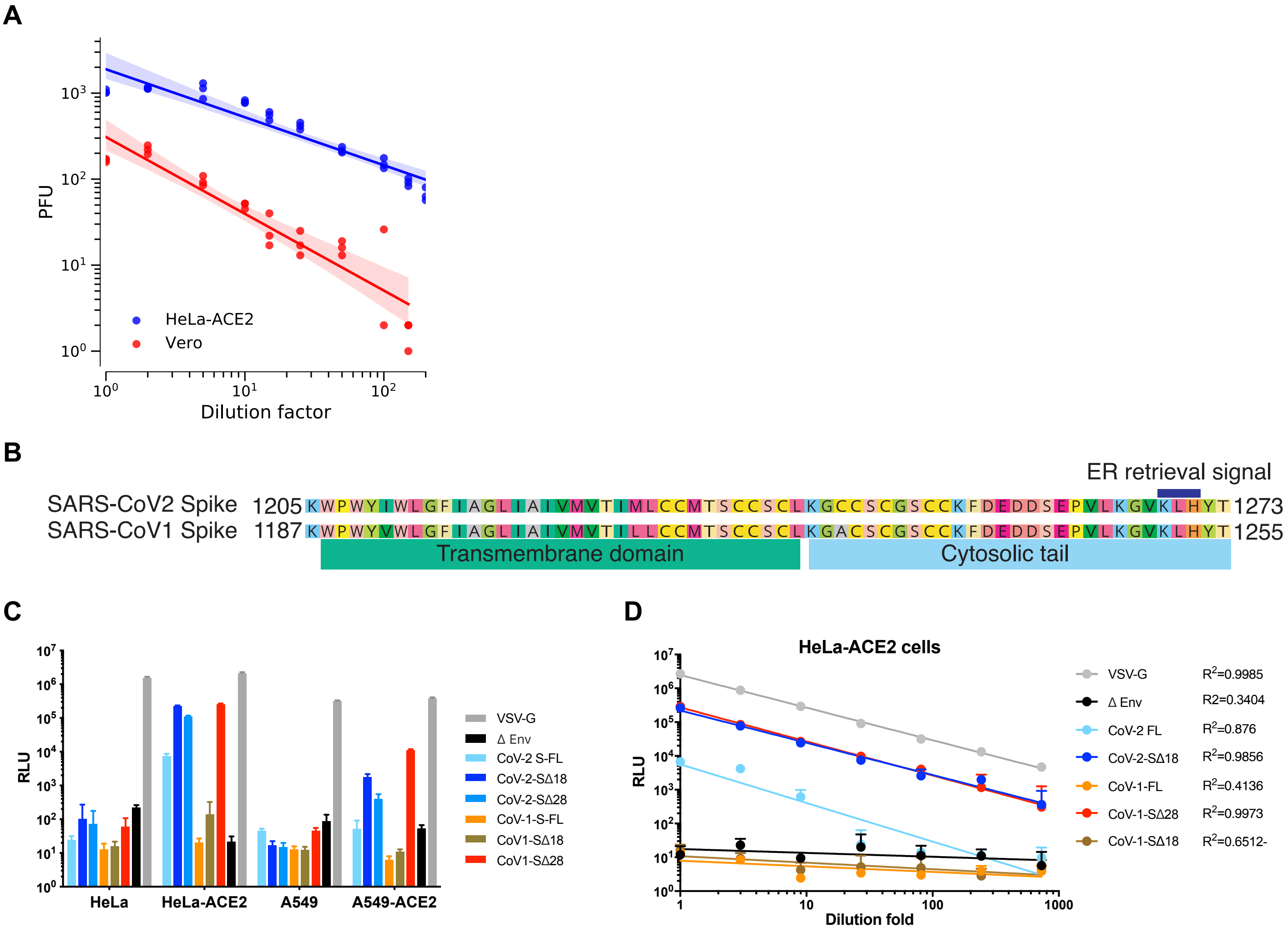

Fig. S1. Neutralization assay development. (**A**) Vero and HeLa-ACE2 cells were infected with serially diluted SARS-CoV-2. Both cell types were plated at 1000 cells/well. The HeLa-ACE2 cell line showed 100% infection at a dilution factor of 2. (**B**) The alignment of SARS-CoV-1 and SARS-CoV-2 transmembrane domain and cytoplasmic tail. (**C**) Comparison of the infectious efficiency of MLV viral particles pseudotyped with the indicated spike (S) proteins: FL indicates full-length spike, D18 and D28 denote C-terminal truncations. Also included were control VSV-G-pseudotyped virions and empty vector control (D Env). Target cells were HeLa cells, A549 cells, HeLa or A549 cells overexpressing human ACE2. After 48h of infection, luciferase expression was assessed. (**D**) Pseudovirions were titrated in HeLa-ACE2 cells in 3-fold serial dilutions. Relative luciferase and fold-dilution are plotted as indicated. Correlation coefficient (R^2) was calculated using linear regression in Prism.

A

Participant ID	ELISA Binding ED_{50}					Cell Surface Binding ID_{50} (%Positive Cells)		Pseudovirus Neutralization ID_{50}			Live Virus Neut. ID_{50}
	SARS-CoV-2 S	SARS-CoV-2 RBD	BSA	SARS-CoV-1 S	SARS-CoV-1 RBD	SARS-CoV-2 Spike	SARS-CoV-1 Spike	SARS-CoV-2	SARS-CoV-1	VSV-G	SARS-CoV-2
CC01	22	<20	<20	<20	<20	<30	75	49	<20	<20	<20
CC04	17036	2233	<20	791	100	29971	4945	2320	90	<20	3382
CC05	<20	<20	<20	<20	<20	<30	<30	<20	<20	<20	<20
CC06	11188	1406	<20	1095	39	297	285	1509	<20	<20	122
CC08	221	20	<20	<20	<20	152	368	<20	<20	<20	35
CC09	83	28	<20	22	<20	158	251	38	<20	<20	55
CC10	552	110	<20	57	<20	281	334	196	<20	<20	538
CC11	113	26	<20	34	<20	395	464	85	<20	<20	108
CC12	307	24	<20	28	<20	572	326	630	<20	<20	478
CC13	117	29	<20	20	<20	378	597	38	<20	<20	122
CC18	41	23	<20	<20	<20	52	102	23	<20	<20	43
CC20	<20	<20	<20	<20	<20	<30	101	<20	<20	<20	<20
CC21	3064	614	<20	434	192	3440	1697	1941	<20	<20	1581
CC22	1202	135	<20	101	<20	2166	1161	406	<20	<20	1478
CC23	186	<20	<20	89	<20	282	416	32	<20	<20	105
CC24	343	51	<20	32	22	458	211	24	<20	<20	106
CC25	1386	560	<20	300	42	1490	668	702	<20	<20	2522

B

	SARS-CoV-2 (Pseudotyped)		SARS-CoV-2 (Live Virus)	
**ID50**	# of Donors	% of Donors	# of Donors	% of Donors
**<500**	12	70	12	70
**500-999**	2	12	1	6
**1000-2500**	3	18	2	12
**>2500**	0	0	2	12

C

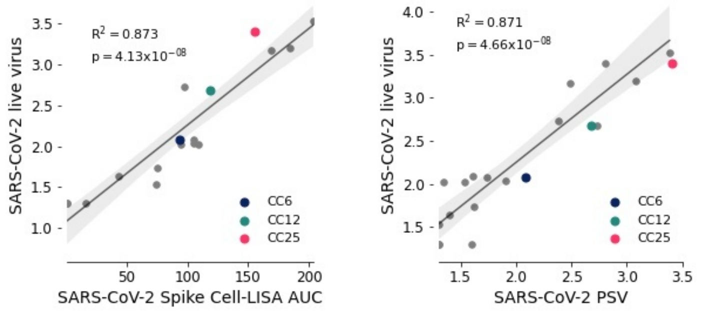

Fig. S2. Evaluation of SARS-2 neutralizing antibody responses in CC participants. Plasma samples collected from 17 COVID-19 Cohort participants were tested for SARS-CoV-1 and CoV-2 pseudotyped and live replicating virus neutralization, cell surface binding (Cell-LISA) to Spike protein and ELISA binding to recombinant S and RBD proteins. (**A**) Plasma titers are indicated. VSV-G was used as negative control. (**B**) Prevalence of potent and weak serum nAb responses to SARS-CoV-2 is compared for pseudotyped and live virus assays for 17 participants, 15 days post symptom onset. (**C)** Correlation between live virus neutralization and Cell-LISA binding assay (left) or pseudovirus neutralization (right) of participant plasma. For both neutralization assays, IC_{50} values are log_{10} transformed before plotting. Linear regression lines are shown. The 95% confidence interval of the regression line is shown in light grey shading and was computed using 1,000 bootstrap resamplings. R^2 and p values are indicated. AUC: Area-under-the-curve. Donors from whom mAbs were isolated are specifically highlighted in dark blue (CC6), pine green (CC12) and hot pink (CC25).

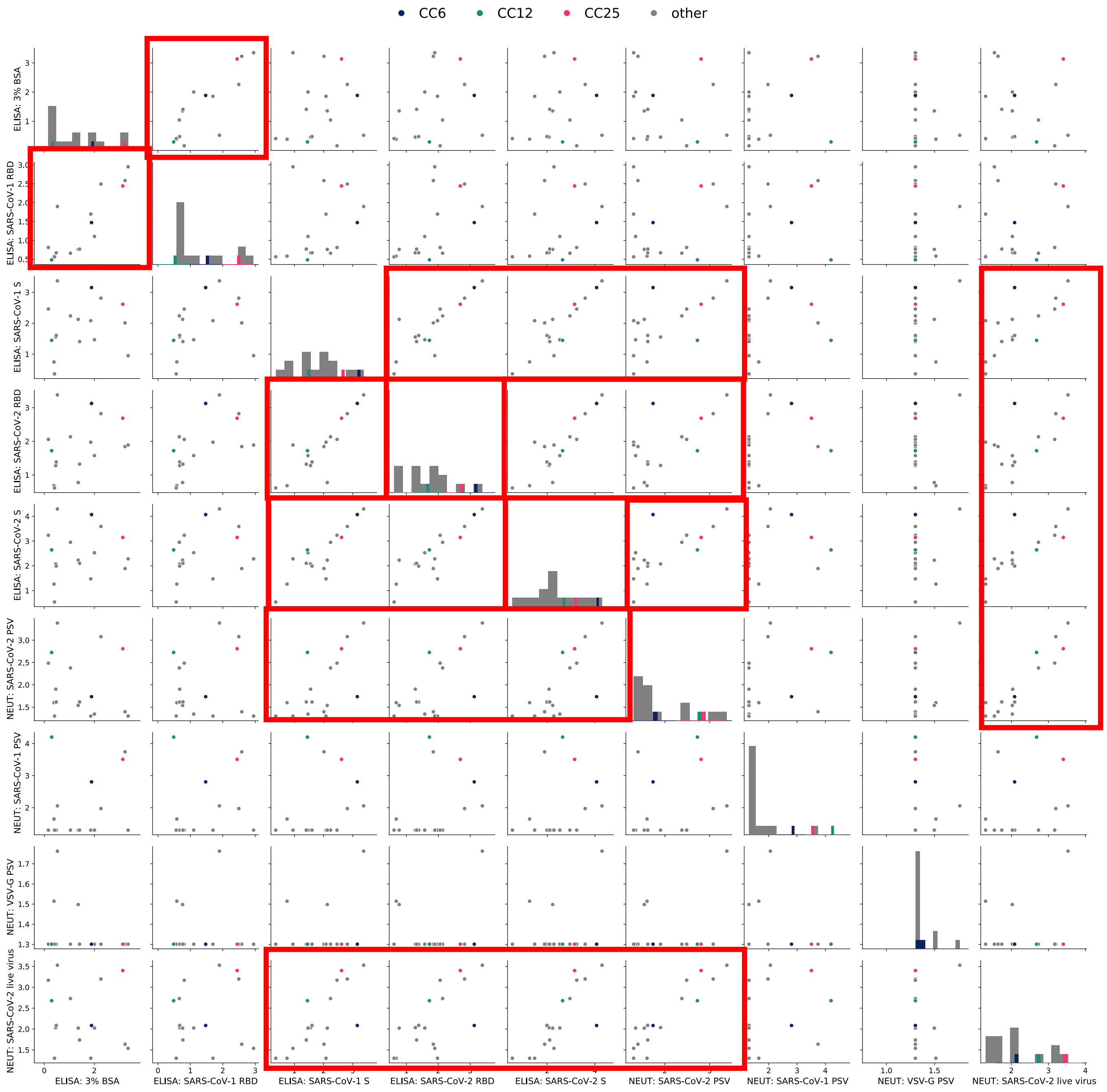

Fig. S3. Evaluation of SARS-2 neutralizing antibody responses in CC participants. Full correlation matrix of individual parameters used to evaluate plasma Ab responses.

A

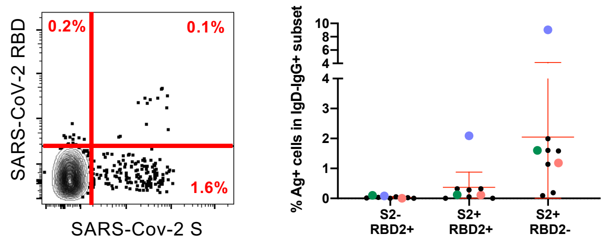

B

Sort	Plate	# wells	Sample#1	Sample#2	HC Global	KC Global	LC Global	PAIRS	HC Cloned	KC Cloned	LC Cloned	#HKL	mAbs Transfected
	1	96	CC7	CC9	59%	36%	40%	44%					
	2	96		CC9	77%	56%	38%	64%					
	3	96		CC9	76%	46%	36%	52%	287	144	101	38	287
	4	96		CC9	80%	48%	40%	59%					
	5	96		CC9	77%	58%	24%	64%					
	6	96	CC10	CC9	93%	53%	39%	80%	39	15	15	2	29
	10	96	CC12	CC11	69%	39%	50%	66%					
	11	96	CC12		83%	50%	41%	73%					
Sort#1	12	96	CC12		75%	43%	46%	74%	273	154	107	28	273
	13	96	CC12		74%	45%	46%	72%					
	14	96	CC12	CC13	73%	42%	44%	71%					
	15	96		CC13	85%	36%	51%	74%					
	7	96	CC10		71%	34%	38%	47%					
	8	96		CC11	79%	42%	46%	77%					
	9	96		CC11	72%	39%	44%	67%					
	16	96		CC13	80%	44%	51%	72%	586	322	223	55	586
	17	88		CC13	73%	38%	32%	63%					
	18	96	CoVID19-		55%	44%	20%	39%					
	1	96		CC12	70%	32%	41%	58%					
Sort#2	2	96		CC12	81%	43%	48%	74%	183	96	106	16	183
	3	96		CC12	69%	39%	33%	58%					
	1	96	CC6		61%	28%	41%	57%					
	2	96	CC6		63%	31%	46%	57%					
Sort#3	3	96	CC6		67%	45%	32%	58%	242	117	145	20	242
	4	96	CC6		69%	26%	47%	60%					
	5	96	CC6		61%	42%	36%	55%					
	1	96	CC25		72%	48%	43%	66%					
	2	96	CC25		77%	50%	41%	70%					
	3	96	CC25		77%	49%	38%	65%					
Sort#4	4	96	CC25	CC6	80%	52%	40%	75%	445	273	96	58	445
	5	96		CC6	79%	47%	49%	69%					
	6	96		CC6	78%	48%	41%	70%					
	7	96		CC6	82%	44%	56%	77%					
**TOTAL**	39	3160	8		2251	1301	1248	2055	2055	1121	793	217	2045

C

	Heavy Chain (H) Colonies	Kappa chain (K) Colonies	Lambda chain (L) Colonies	H+K+L All Colonies
Total Colonies Screened	819	819	819	2457
Off-frame insert	2	2	0	0.2%
IMGT: No Results	45	4	9	2.4%
IMGT: Unproductive	33	20	12	2.6%
IMGT: No rearrangement found	7	0	0	0.3%
Total Cloning Reactions	273	273	273	819
Overall Cloning Failures	12	18	11	5.0%
Total Cloned Pairs		273		273
Predicted Unproductive Pairs		37		13.6%

Fig. S4. SARS-CoV-2 mAb isolation, cloning and expression summary. (A) FACS frequency of memory (CD3-CD19+IgD-IgG+) B cells binding to SARS-CoV-2 S-protein (S2+) and or RBD subunit (RBD2+) in COVID-19 individuals. (Left) A representative fluorescence dot plot is shown for CC12. (Right) Frequencies for all donors are plotted. Average and SD are indicated with red lines. (**B**) The following statistics are tabulated **:** number of SARS-CoV-2 specific B-cells sorted for each donor in each experiment, efficiency of heavy and light chains PCR recovery (% of PCR positive wells, color gradient with warmer color indicating higher efficiency), number of heavy and light chains amplicons cloned; number of and H+L pairs transfected (green) for high-throughput functional screening. (**C**) Three individual colonies were picked from 273 paired H+L cloning reactions, send for sequencing and analyzed using IMGT V-Quest. Cloning efficiency statistics are indicated.

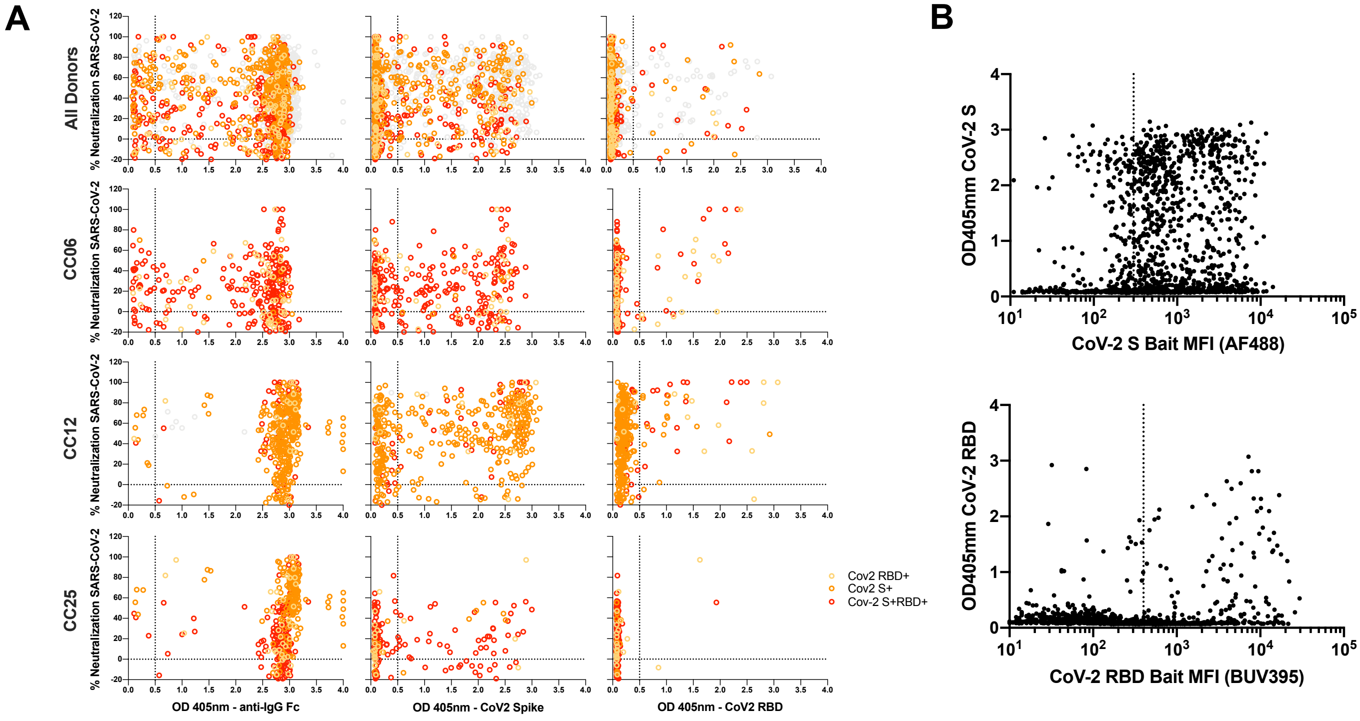

Fig. S5. Functional screening of Ab H+L pairs rescued from SARS-CoV-2-specific single B-cell sorting. (**A**) Cloned H+L chain pairs isolated from SARS-2 specific single B-cells were transfected into a high efficiency expression cell line. Small-scale culture supernatants were harvested at day5-post transfection and evaluated for the presence of IgG, binding to recombinant SARS-CoV-2 S-protein and RBD subunit as well as for pseudotyped SARS-CoV-2 neutralization. ELISA considered positive when OD405nm was > 0.5 (dotted line). Results are plotted to show the proportion of expressed, binding and neutralizing pairs. (**B**) Correlation between ELISA binding signal (OD 405nm) and corresponding sorted cell staining level (MFI) for each antigenic bait (SARS-CoV-2 S-protein or RBD).

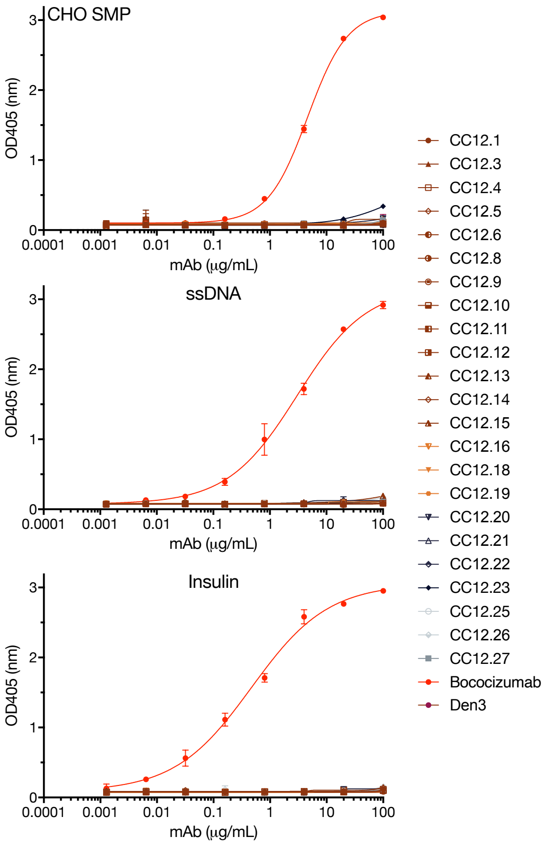

Fig S6. Evaluation of SARS-CoV-2 specific mAbs for polyreactivity and autoreactivity. Antibodies were tested by ELISA for binding to several polyspecificity reagents (PSR): CHO-cell soluble membrane protein extracts (SMP), single stranded DNA (ssDNA) and Insulin.

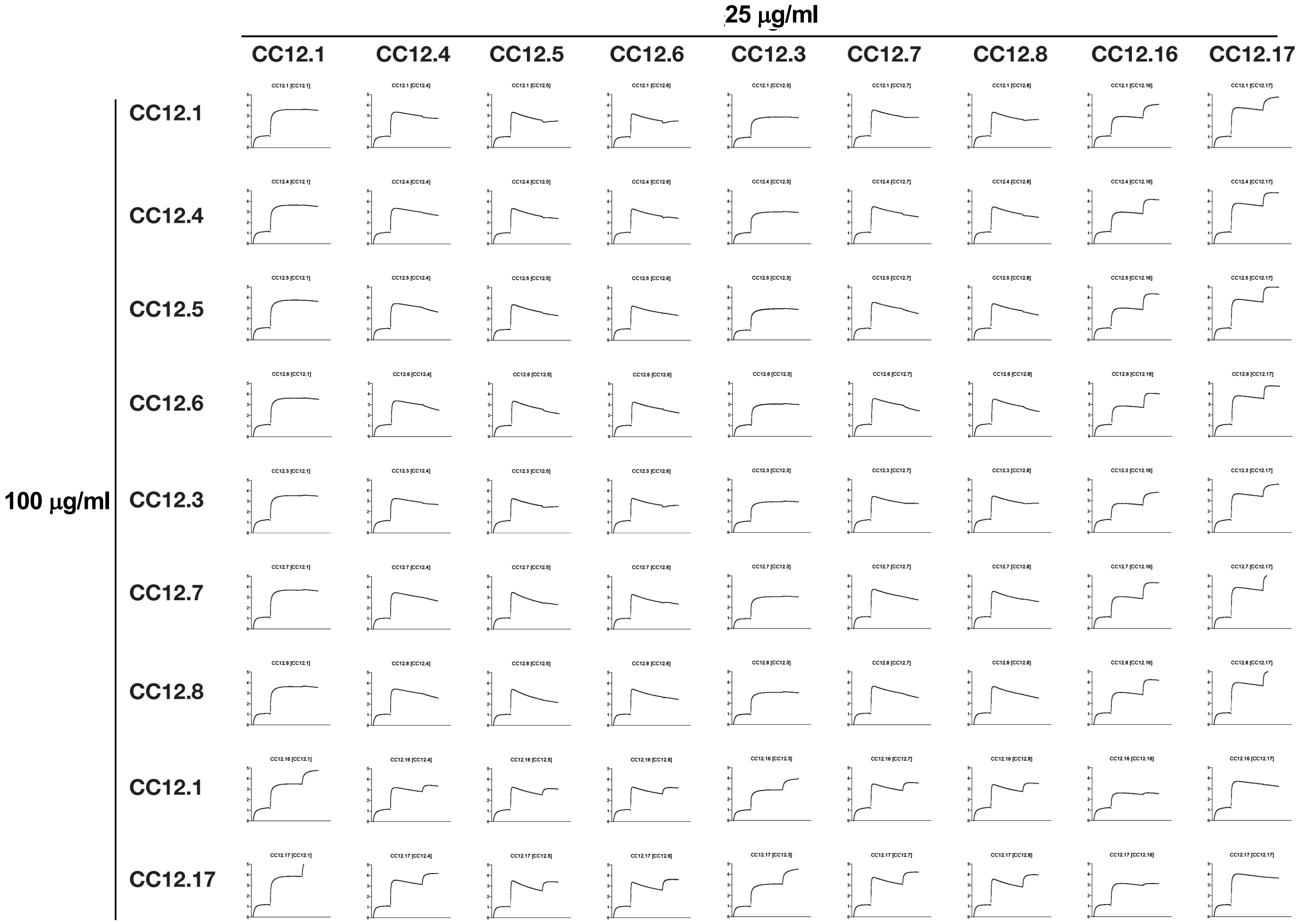

Fig. S7A: Monoclonal antibodies were evaluated for epitope competition using an Octet RED384 platform. His-tagged RBD was captured using an anti-Penta-HIS biosensor and indicated mAbs at a concentration of 100 µg/ml are first incubated for 10 min followed by incubation with 25 µg/ml of competing antibodies for 5 min.

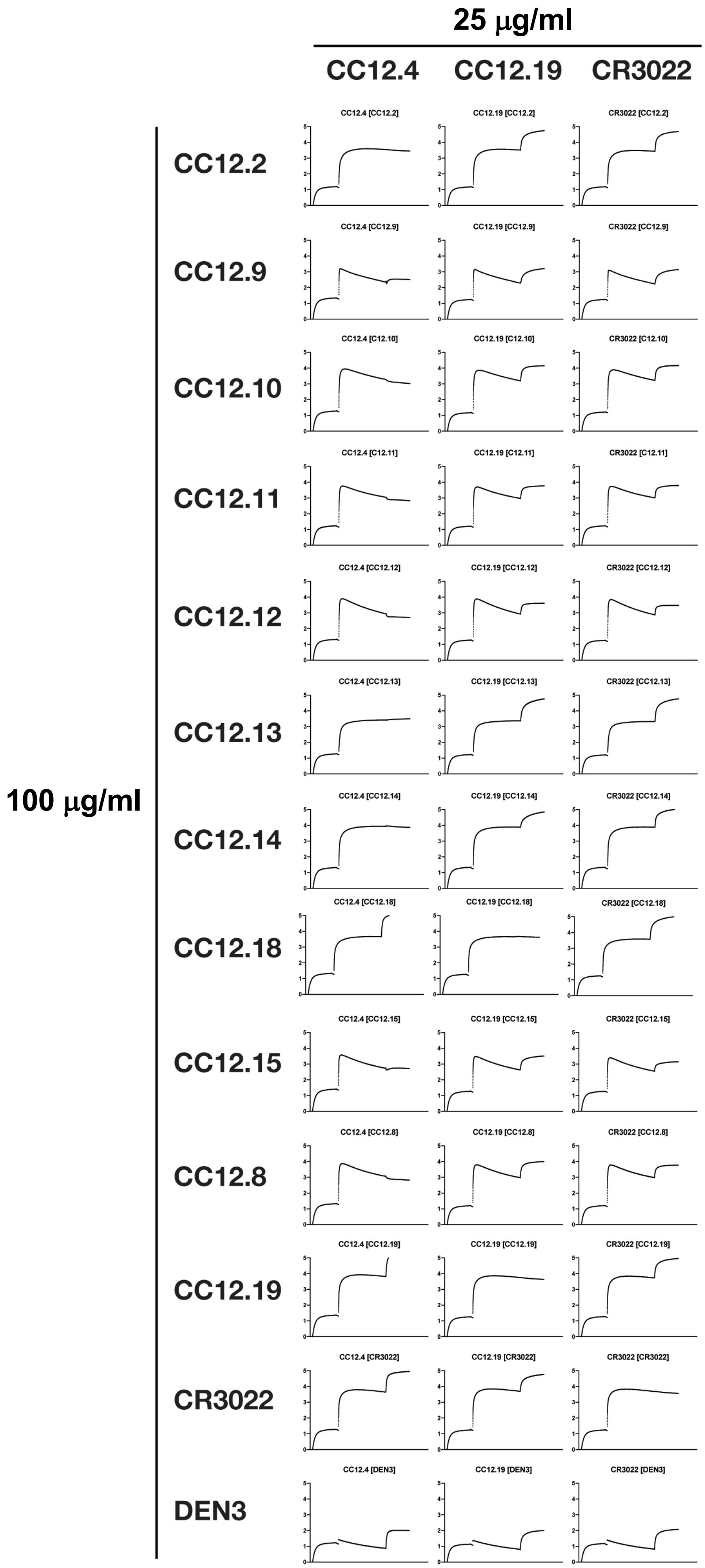

Fig. S7B: Monoclonal antibodies were evaluated for epitope competition using an Octet RED384 platform. His-tagged RBD was captured using an anti-Penta-HIS biosensor and indicated mAbs at a concentration of 100 μg/ml are first incubated for 10 min followed by incubation with 25 μg/ml of competing antibodies for 5 min.

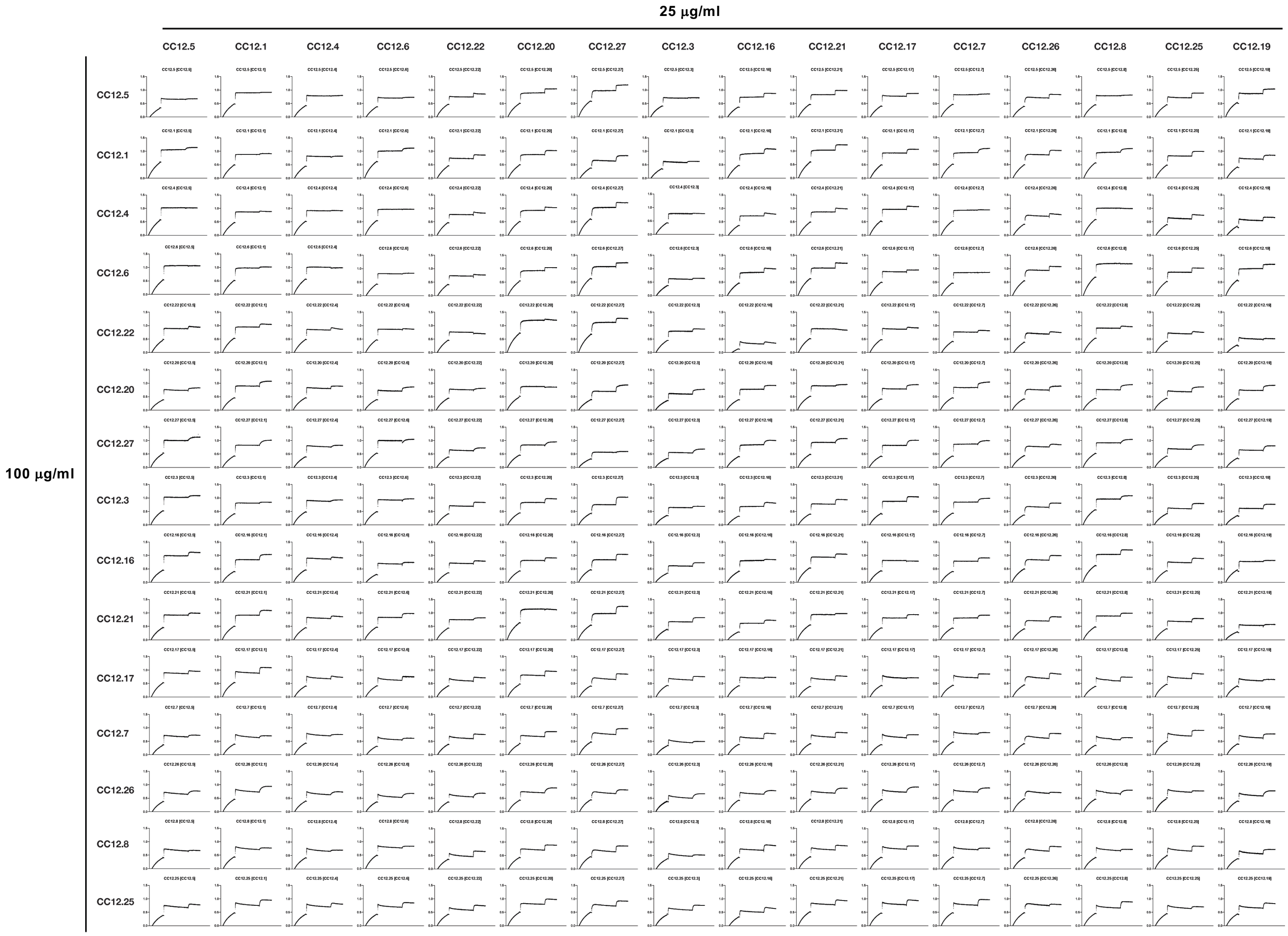

Fig. S7C: Monoclonal antibodies were evaluated for epitope competition using an Octet RED384 platform. His-tagged RBD was captured using an anti-Penta-HIS biosensor and indicated mAbs at a concentration of 100 μg/ml are first incubated for 10 min followed by incubation with 25 μg/ml of competing antibodies for 5 min.

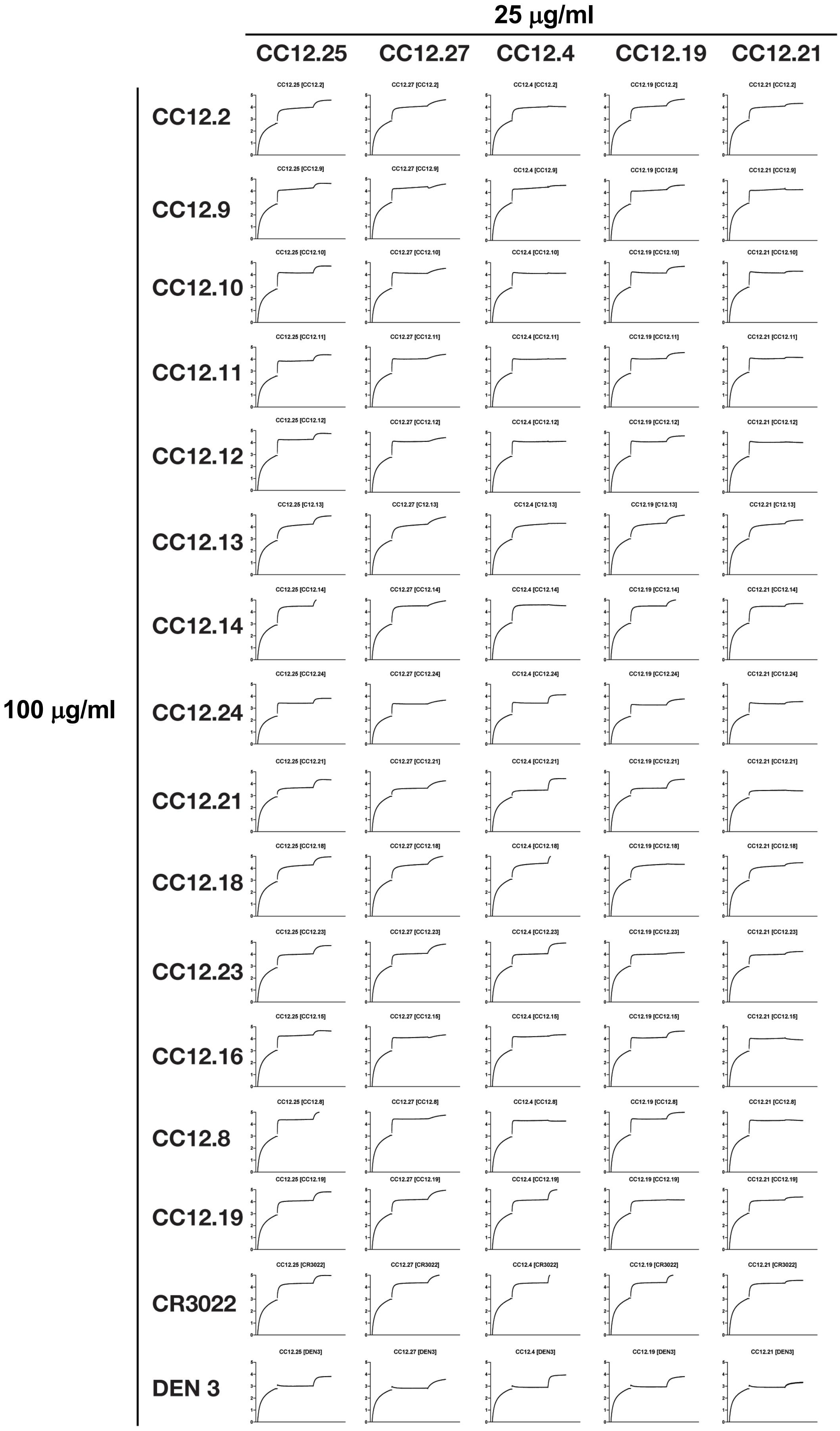

Fig. S7D: Monoclonal antibodies were evaluated for epitope competition using an Octet RED384 platform. His-tagged RBD was captured using an anti-Penta-HIS biosensor and indicated mAbs at a concentration of 100 μg/ml are first incubated for 10 min followed by incubation with 25 μg/ml of competing antibodies for 5 min.

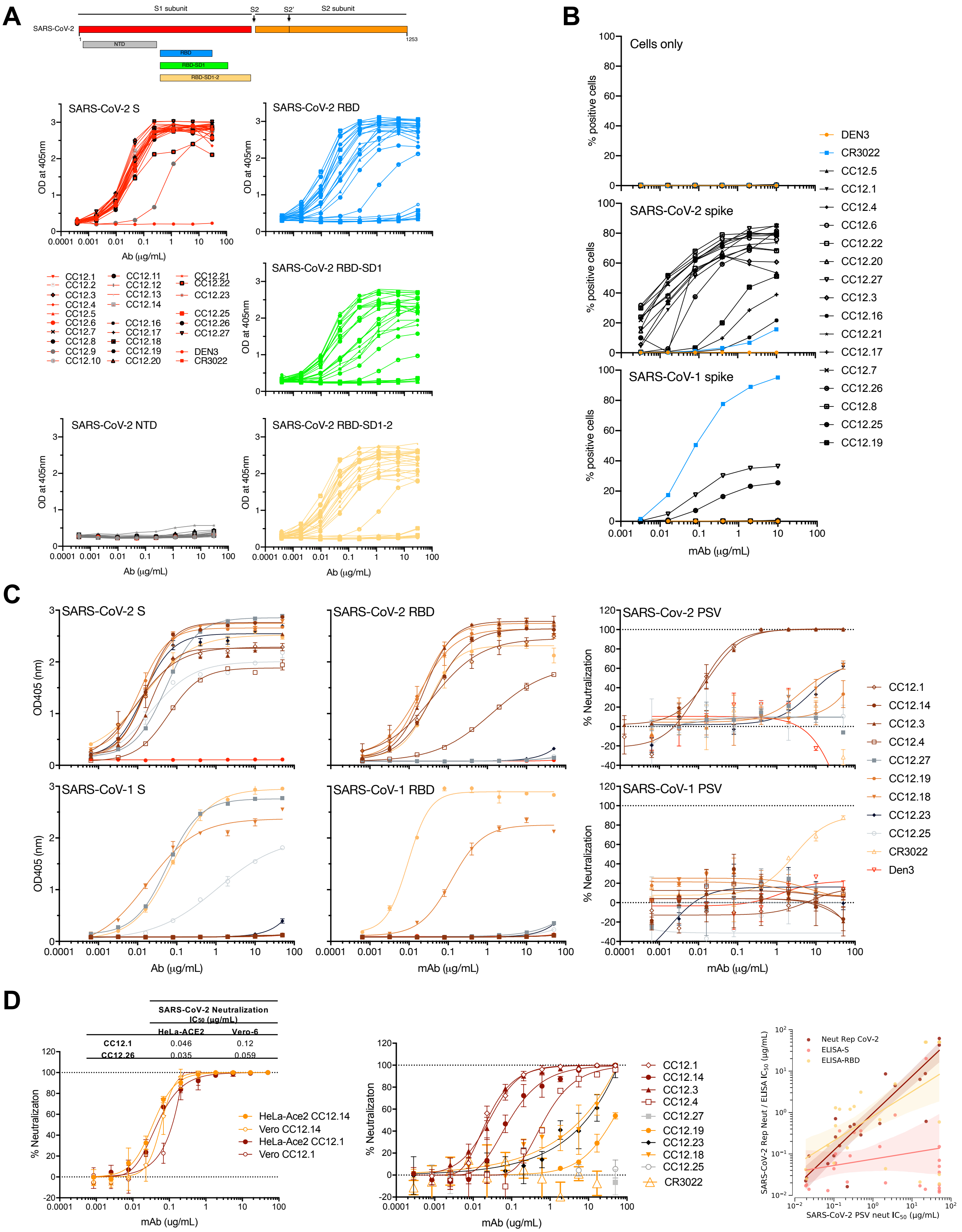

Fig S8. Functional characterization of SARS-CoV-2 specific mAbs selected from the HTP screening. Antibodies were tested for **(A)** ELISA for binding to several truncated versions of the recombinant SARS-CoV-1 and SARS-CoV-2 S proteins, (**B**) cell surface binding to SARS-CoV-1 and CoV-2 Spike, and for neutralization of (**C**) pseudotyped and (**D**) replicating SARS-CoV-2 on HeLa-ACE2 or Vero (middle plot only). **(E)** ACE2 binding inhibition was performed by flow cytometry. Purified mAbs were mixed with biotinylated SARS-CoV-2 S or RBD proteins at a molar ratio of 4:1 before adding to HeLa-ACE2 cells. Streptavidin-AF647 was used to detect the relative intensity of cell surface ACE2 binding biotinylated SARS-CoV-2 S or RBD proteins. SARS-CoV-2 S or RBD-AF647 mean fluorescence intensity (MFI) was determined from the gate of singlet and PI negative cells. CR3022 and Den3 were used as negative controls. HeLa and HeLa-ACE2 cells stained with SARS-CoV-2 S or RBD alone were used as background and positive control separately.

E

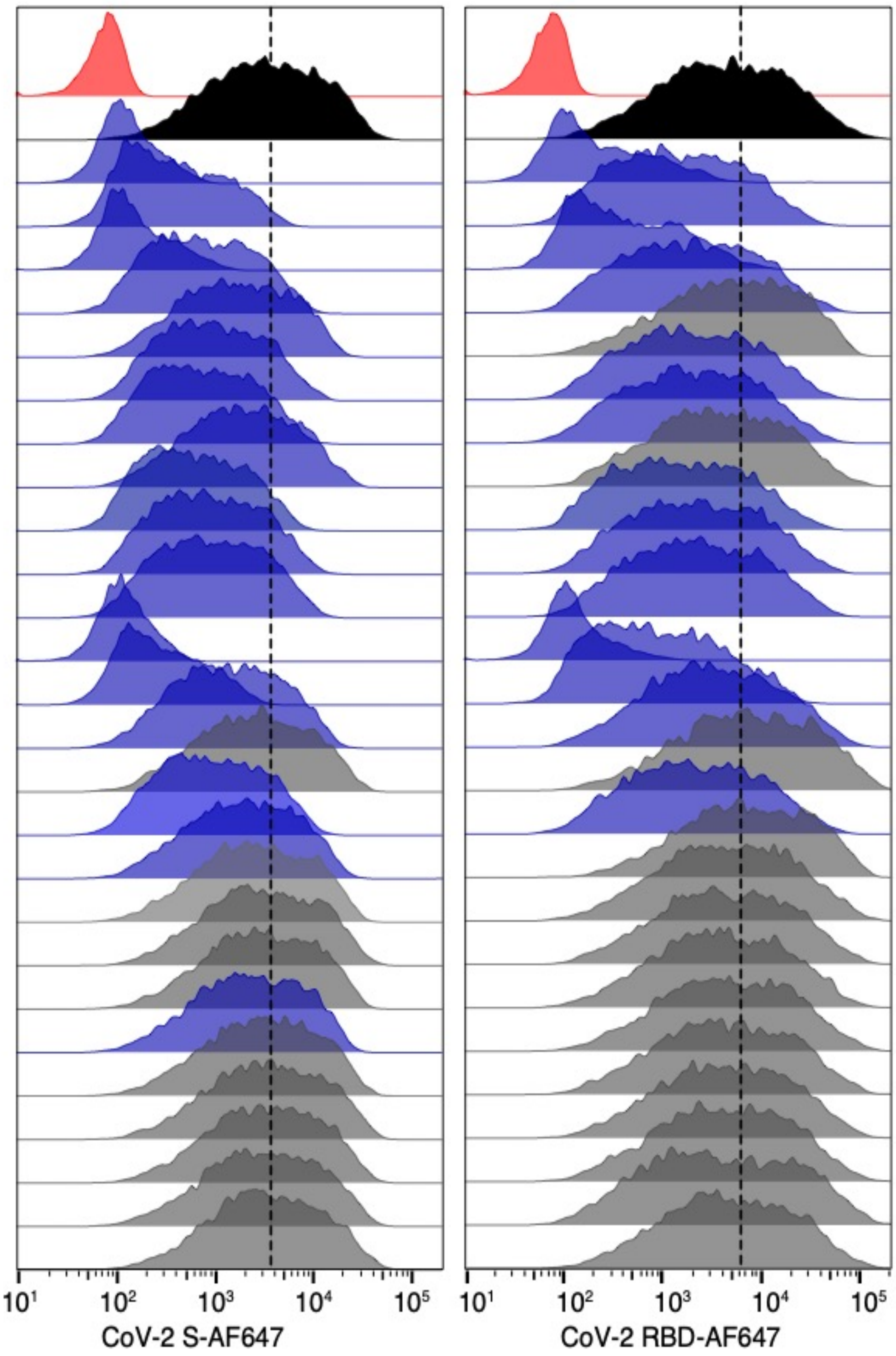

mAbs	MFI of CoV-2 S	MFI of CoV-2 RBD
Background	80.0	74.5
Probe alone	6502	10817
CC12.1	165	528
CC12.2	731	3682
CC12.3	214	982
CC12.4	1430	5194
CC12.5	3155	11672
CC12.6	1756	5167
CC12.8	1369	5105
CC12.9	3765	9714
CC12.10	1211	4036
CC12.11	1681	5614
CC12.12	1787	5738
CC12.13	145	178
CC12.14	463	2228
CC12.15	2736	8718
CC12.16	4945	18054
CC12.18	1792	5711
CC12.19	3726	16358
CC12.20	4959	11677
CC12.21	5869	11140
CC12.22	5504	10020
CC12.23	4189	11601
CC12.25	5834	11207
CC12.26	6660	11004
CC12.27	6219	10918
CR3022	6077	12763
Den3	6567	11681

Fig S8. Functional characterization of SARS-CoV-2 specific mAbs selected from the HTP screening. Antibodies were tested for (**A)** ELISA for binding to several truncated versions of the recombinant SARS-CoV-1 and SARS-CoV-2 S protein, (**B**) cell surface binding to SARS-CoV-1 and CoV-2 Spike, and for neutralization of (**C**) pseudotyped and (**D**) replicating SARS-CoV-2 on HeLa-ACE2 or Vero (middle plot only). **(E)** ACE2 binding inhibition was performed by flow cytometry. Purified mAbs were mixed with biotinylated SARS-CoV-2 S or RBD proteins at a molar ratio of 4:1 before adding to HeLa-ACE2 cells. Streptavidin-AF647 was used to detect the relative intensity of cell surface ACE2 binding biotinylated SARS-CoV-2 S or RBD proteins. SARS-CoV-2 S or RBD-AF647 mean fluorescence intensity (MFI) was determined from the gate of singlet and PI negative cells. CR3022 and Den3 were used as negative control. HeLa and HeLa-ACE2 cells stained with SARS-CoV-2 S or RBD alone were used as background and positive control separately.

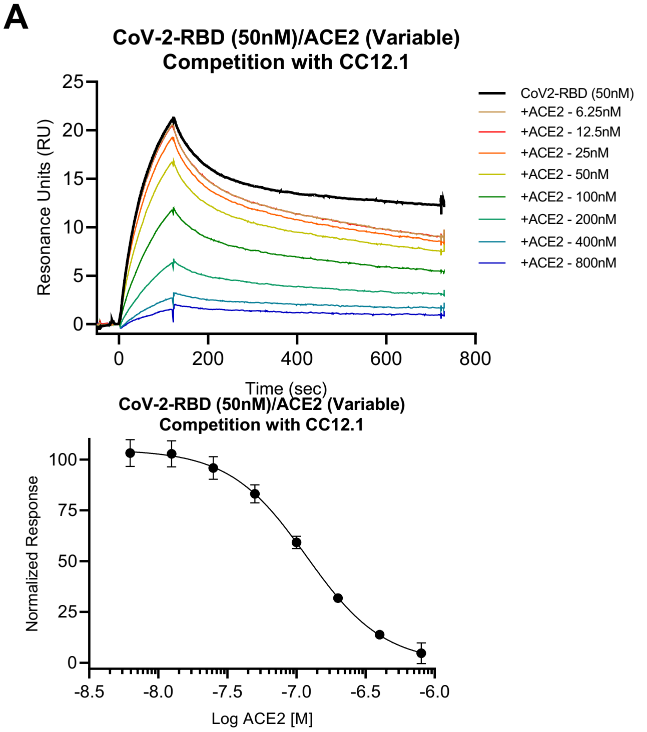

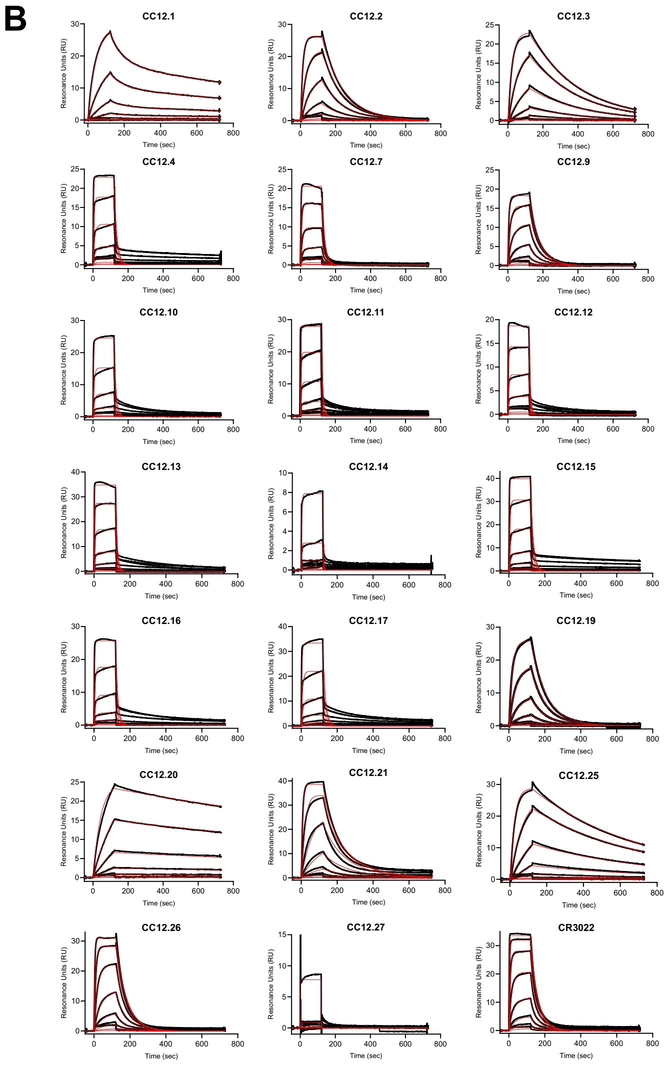

C

mAb ID	Method	Analyte	Fit Model	ka [1/Ms]	kd [1/s]	KD [M]
CC12.1	Fc-Capture	CoV-2-RBD	1:1 Binding	6.95E+05	4.12E-03	5.92E-09
CC12.2	Fc-Capture	CoV-2-RBD	1:1 Binding	1.48E+05	8.03E-03	5.43E-08
CC12.3	Fc-Capture	CoV-2-RBD	1:1 Binding	3.99E+05	3.42E-03	8.59E-09
CC12.4	Fc-Capture	CoV-2-RBD	1:1 Binding	3.88E+05	6.60E-02	1.70E-07
CC12.5	Fc-Capture	CoV-2-RBD	1:1 Binding	-	-	ND
CC12.6	Fc-Capture	CoV-2-RBD	1:1 Binding	-	-	ND
CC12.7	Fc-Capture	CoV-2-RBD	1:1 Binding	3.80E+05	6.03E-02	1.59E-07
CC12.8	Fc-Capture	CoV-2-RBD	1:1 Binding	-	-	ND
CC12.9	Fc-Capture	CoV-2-RBD	1:1 Binding	3.64E+05	1.70E-02	4.66E-08
CC12.10	Fc-Capture	CoV-2-RBD	1:1 Binding	4.36E+05	9.61E-02	2.20E-07
CC12.11	Fc-Capture	CoV-2-RBD	1:1 Binding	4.93E+05	1.26E-01	2.55E-07
CC12.12	Fc-Capture	CoV-2-RBD	1:1 Binding	7.82E+05	1.43E-01	1.83E-07
CC12.13	Fc-Capture	CoV-2-RBD	1:1 Binding	7.73E+05	1.20E-01	1.55E-07
CC12.14	Fc-Capture	CoV-2-RBD	1:1 Binding	5.94E+04	3.12E-01	>5.00E-07
CC12.15	Fc-Capture	CoV-2-RBD	1:1 Binding	7.86E+05	6.91E-02	8.79E-08
CC12.16	Fc-Capture	CoV-2-RBD	1:1 Binding	5.75E+05	8.43E-02	1.47E-07
CC12.17	Fc-Capture	CoV-2-RBD	1:1 Binding	4.04E+05	7.07E-02	1.75E-07
CC12.18	Fc-Capture	CoV-2-RBD	1:1 Binding	-	-	ND
CC12.19	Fc-Capture	CoV-2-RBD	1:1 Binding	4.43E+05	1.15E-02	2.60E-08
CC12.20	Fc-Capture	CoV-2-RBD	1:1 Binding	1.40E+05	3.64E-04	2.60E-09
CC12.21	Fc-Capture	CoV-2-RBD	1:1 Binding	4.35E+05	1.57E-02	3.62E-08
CC12.22	Fc-Capture	CoV-2-RBD	1:1 Binding	-	-	ND
CC12.23	Fc-Capture	CoV-2-RBD	1:1 Binding	-	-	ND
CC12.24	Fc-Capture	CoV-2-RBD	1:1 Binding	-	-	ND
CC12.25	Fc-Capture	CoV-2-RBD	1:1 Binding	4.28E+05	1.66E-03	3.88E-09
CC12.26	Fc-Capture	CoV-2-RBD	1:1 Binding	6.36E+05	1.70E-02	2.68E-08
CC12.27	Fc-Capture	CoV-2-RBD	1:1 Binding	4.45E+03	1.64E+00	>1.00E-6
CR3022	Fc-Capture	CoV-2-RBD	1:1 Binding	1.03E+06	2.54E-02	2.47E-08

Fig. S9: SARS-CoV-2 nAb affinities. (**A**) CC12.1 binding inhibits the interaction of SARS-CoV-2-RBD with ACE2. (Top) Representative data from a SPR competition experiment in which the effect of varying concentrations of ACE2 on the interaction of 50 nM SARS-CoV-2-RBD with CC12.1 was examined. A legend showing the identity of each sensorgram is *inset*, in which the ACE2 injection alone was subtracted from the SARS-CoV-2-RBD + ACE2 injection series. The residual SARS-CoV-2-RBD binding in the presence of multiple ACE2 concentrations is shown in varying colors, while the sensorgram for the same concentration of SARS-CoV-2-RBD in the absence of ACE2 is shown as the darkest black line. (Bottom) Residual SARS-CoV-2-RBD binding in the presence of various concentrations of ACE2, normalized to 50 nM CoV-2-RBD (100%) and buffer (0%), and fit to a dose-response curve (IC_{50} = 120 nM). (**B**) SPR sensorgrams for SARS-CoV-2-RBD binding. Recombinant antibodies were captured via Fc-capture to an anti-human IgG (Fc) antibody and varying concentrations of SARS-CoV-2-RBD were injected using a multi-cycle method. Representative sensorgrams in resonance units (RUs) plotted against time of injection are shown. Black lines are the experimental trace obtained from the SPR experiments and red are the best global fits (1:1 Langmuir binding model) to the data used to calculate the association (ka) and dissociation (kd) rate constants. (**C**) Summarized results of SARS-CoV-2-RBD binding to antibodies via a Fc-capture, multi-cycle method. Association and dissociation rate constants calculated through a 1:1 Langmuir binding model using the BIAevaluation software. ND = not determined.

mAb name	Predicted Unique Epitope	ELISA IC$_{50}$ (µg/mL)				Poly-reactivity	PSV Neutralization IC$_{50}$ (µg/mL)			Live virus SARS-CoV-2
		SARS-CoV-2 S	SARS-CoV-2 RBD	SARS-CoV-1 S	SARS-CoV-1 RBD		SARS-CoV-1	SARS-CoV-2	MPN	
**CC12.1**	RBD-A	0.017	0.042	>50	>50	None	>50	0.019	100%	0.022
**CC12.2**	RBD-A	0.021	0.14	>50	>50	None	>50	0.22	100%	0.016
**CC12.3**	RBD-A	0.018	0.025	>50	>50	None	>50	0.018	100%	0.026
**CC12.4**	RBD-A	0.062	1.3	>50	>50	None	>50	0.11	100%	0.71
**CC12.5**	RBD-A	0.33	1.4	>50	>50	None	>50	0.33	100%	0.52
**CC12.6**	RBD-A	0.36	3.1	>50	>50	None	>50	0.49	100%	0.29
**CC12.7**	RBD-A	0.091	1.2	>50	>50	None	>50	0.26	89%	0.46
**CC12.8**	RBD-A	0.10	0.48	>50	>50	None	>50	0.11	100%	0.26
**CC12.9**	RBD-A	20	>50	>50	>50	None	>50	23	60%	0.61
**CC12.10**	RBD-A	0.013	0.16	>50	>50	None	>50	0.07	95%	0.058
**CC12.11**	RBD-A	0.36	1.2	>50	>50	None	>50	0.14	85%	0.16
**CC12.12**	RBD-A	0.15	0.67	>50	>50	ND	>50	1.5	99%	0.54
**CC12.13**	RBD-A	0.044	0.058	>50	>50	None	>50	0.10	100%	0.090
**CC12.14**	RBD-A	0.014	0.039	>50	>50	ND	>50	0.023	100%	0.089
**CC12.15**	RBD-A	0.87	4.9	>50	>50	None	>50	3.7	65%	4.5
**CC12.16**	RBD-B	0.073	0.59	>50	>50	None	>50	>50	21%	61
**CC12.17**	RBD-B	0.020	0.044	>50	>50	None	>50	2.1	91%	8.6
**CC12.18**	RBD-B	0.017	0.021	0.018	2.1	None	>50	16	66%	6.3
**CC12.19**	RBD-B	0.012	0.019	>50	>50	None	>50	>50	50%	43
**CC12.20**	SPIKE-A	0.036	>50	>50	>50	None	>50	>50		>50
**CC12.21**	SPIKE-A	0.0050	>50	>50	>50	None	>50	>50		>50
**CC12.22**	SPIKE-A	>50	>50	>50	>50	None	>50	>50	41%	>50
**CC12.23**	SPIKE-A	0.014	>50	>50	>50	None	>50	22	64%	8.7
**CC12.24**	ND	0.013	>50	>50	>50	None	>50	>50		ND
**CC12.25**	SPIKE-B	0.018	>50	1.1	>50	None	>50	>50		>50
**CC12.26**	SPIKE-B	0.15	>50	>50	>50	None	>50	>50		>50
**CC12.27**	SPIKE-C	0.053	>50	0.057	>50	None	>50	>50		>50
**CR3022**	RBD-C	0.015	0.032	2.9	2.7	None	3.0	>50		
**Den3**		>50	>50	>50	>50	None	>50	>50		

Fig. S10: SARS-CoV-2 nAb functional summary. SARS-Cov-1 and SARS-CoV-2 binding affinities and neutralization potency of the indicated SARS-CoV-2 specific mAbs isolated from CC12 are tabulated. Neutralization was tested against pseudotyped (PSV) and live replicating SARS-CoV-1 and SARS-CoV-2 viruses. MPN: Maximum Neutralization Plateau.

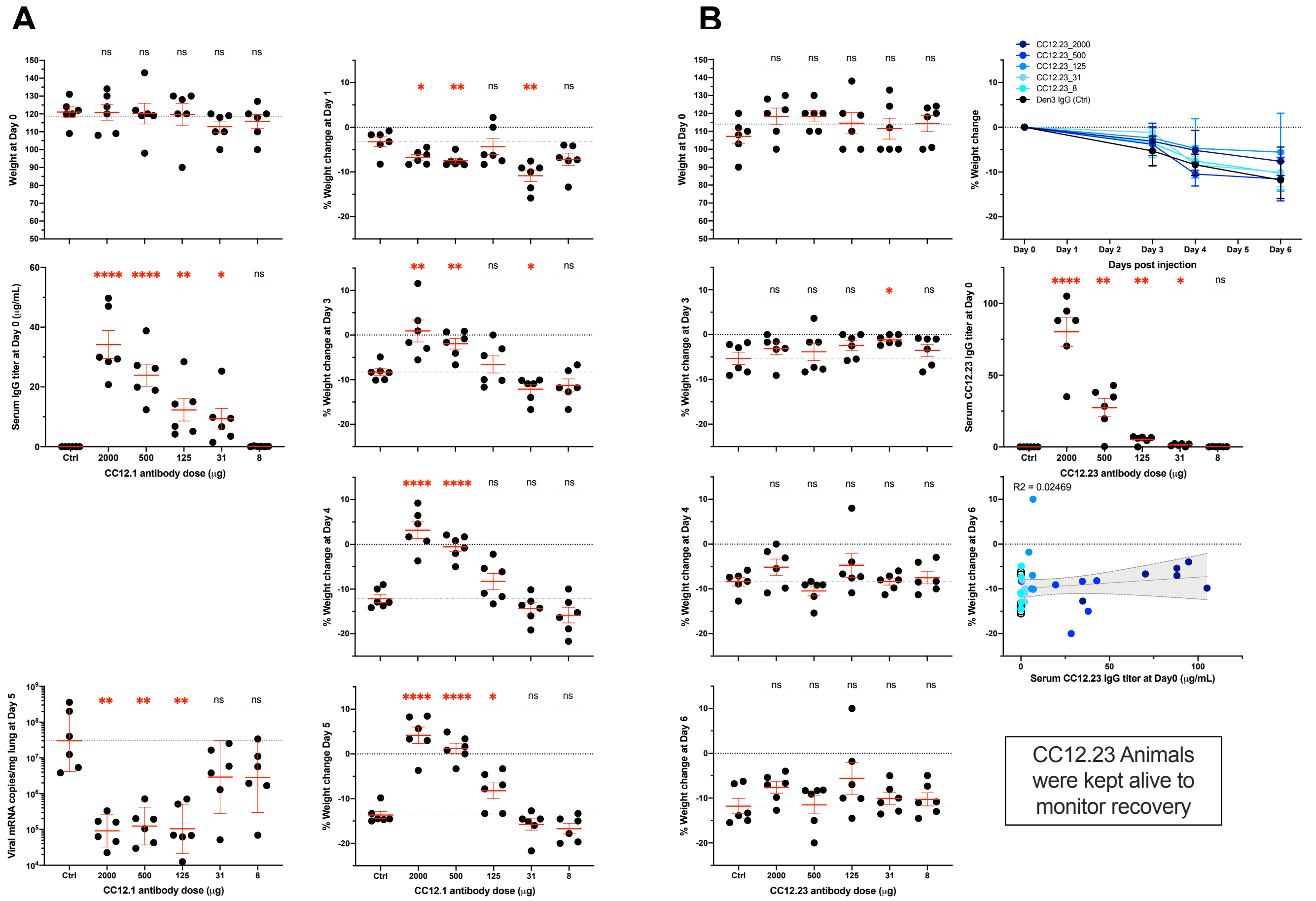

Fig.S11: Animal protection studies. Syrian hamsters received various doses of SARS-CoV-2-specific human mAbs CC12.1 or CC12.23 or 2 mg dengue-specific human mAb Den3 i.p 12h before i.n. challenge with SARS-CoV-2. (**A**) CC12.1 protection experiment. From top to bottom, left to right: Weights of animals at time of challenge (Day 0); CC12.1 serum concentration in each animal as measured by ELISA at time of challenge (12h post administration, Day 0); Viral load in lung tissue for each animal at day5-post challenge; linear correlation between serum human IgG concentration at time of termination (Day 5) and % weight loss at day 5 (95% confidence intervals indicated in grey shade, R-square value is also indicated); Weight change (%) in each animal at Day 1,3,4 and 5 post challenge. (**B**) CC12.23 protection experiment. From top to bottom, left to right: Weights of animals at time of challenge (Day 0); Weight change (%) in each animal at Day 3, 4 and 5 post challenge (Day 0); average weight loss in each group over time.; linear correlation between serum human IgG concentration at time of challenge (Day 0) and % weight loss at Day 5 (95% confidence intervals indicated in grey shade, R-square value is also indicated). Significance of the difference between the groups was evaluated with Mann-Whitney U-tests using a 95% confidence interval. P-values are indicated (ns: non-significant; (*) <0.0332; (**) <0.0021; (***) <0.0002; (****) <0.00001).

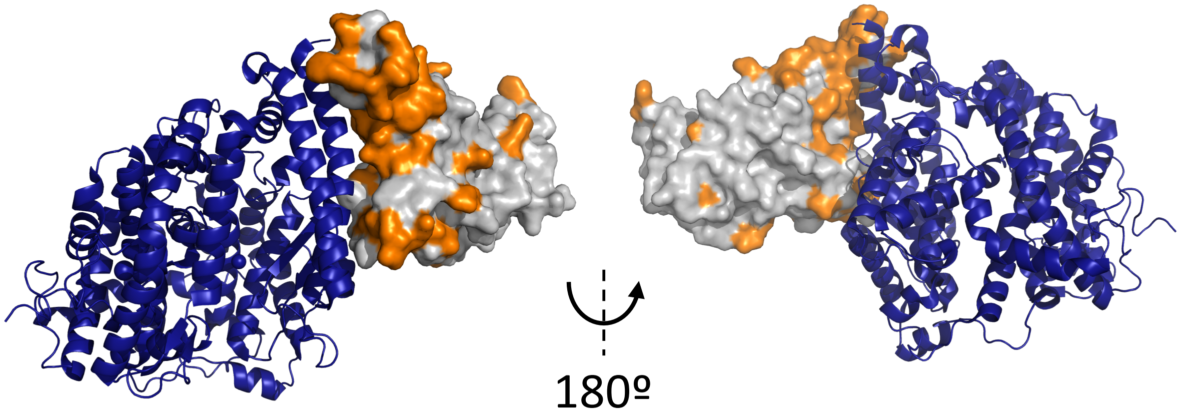

Fig.S12: Crystal structure of SARS-CoV-2 RBD bound to human ACE2 (adapted from PDBID 6M0J). ACE2 is represented as blue ribbons and RBD is surface rendered, with mutations from SARS-CoV-1 highlighted in orange.

SUPPLEMENTARY REFERENCES

16. Kirchdoerfer RN, Cottrell CA, Wang N, Pallesen J, Yassine HM, Turner HL, et al. Pre-fusion structure of a human coronavirus spike protein. Nature. 2016;531: 118–121.
17. Wrapp D, Wang N, Corbett KS, Goldsmith JA, Hsieh C-L, Abiona O, et al. Cryo-EM structure of the 2019-nCoV spike in the prefusion conformation. Science. 2020;367: 1260–1263.
18. Walker LM, Phogat SK, Chan-Hui P-Y, Wagner D, Phung P, Goss JL, et al. Broad and potent neutralizing antibodies from an African donor reveal a new HIV-1 vaccine target. Science. 2009;326: 285–289.
19. Wu X, Yang Z-Y, Li Y, Hogerkorp C-M, Schief WR, Seaman MS, et al. Rational design of envelope identifies broadly neutralizing human monoclonal antibodies to HIV-1. Science. 2010;329: 856–861.
20. Sok D, van Gils MJ, Pauthner M, Julien J-P, Saye-Francisco KL, Hsueh J, et al. Recombinant HIV envelope trimer selects for quaternary-dependent antibodies targeting the trimer apex. Proc Natl Acad Sci U S A. 2014;111: 17624–17629.
21. Tiller T, Meffre E, Yurasov S, Tsuiji M, Nussenzweig MC, Wardemann H. Efficient generation of monoclonal antibodies from single human B cells by single cell RT-PCR and expression vector cloning. J Immunol Methods. 2008;329: 112–124.
22. Doria-Rose NA, Schramm CA, Gorman J, Moore PL, Bhiman JN, DeKosky BJ, et al. Developmental pathway for potent V1V2-directed HIV-neutralizing antibodies. Nature. 2014;509: 55–62.
23. Lefranc M-P, Giudicelli V, Ginestoux C, Jabado-Michaloud J, Folch G, Bellahcene F, et al. IMGT, the international ImMunoGeneTics information system. Nucleic Acids Res. 2009;37: D1006–12.
24. Ricklin D, Tzekou A, Garcia BL, Hammel M, McWhorter WJ, Sfyroera G, et al. A molecular insight into complement evasion by the staphylococcal complement inhibitor protein family. J Immunol. 2009;183: 2565–2574.
25. Woehl JL, Stapels DAC, Garcia BL, Ramyar KX, Keightley A, Ruyken M, et al. The extracellular adherence protein from Staphylococcus aureus inhibits the classical and lectin pathways of complement by blocking formation of the C3 proconvertase. J Immunol. 2014;193: 6161–6171.
